# Deletion of *Atg22* gene contributes to reduce programmed cell death induced by acetic acid stress in *Saccharomyces cerevisiae*

**DOI:** 10.1186/s13068-019-1638-x

**Published:** 2019-12-27

**Authors:** Jingjin Hu, Yachen Dong, Wei Wang, Wei Zhang, Hanghang Lou, Qihe Chen

**Affiliations:** 1 Department of Food Science and Nutrition, Key Laboratory for Food Microbial Technology of Zhejiang Province, Zhejiang University, Hangzhou, 310058 China; 2 Department of Cardiovascular & Metabolic Sciences, The Lerner Research Institute, Cleveland Clinic, Cleveland, OH USA; 3 Institute of Quality and Standard for Agriculture Products, Zhejiang Academy of Agricultural Sciences, Hangzhou, 310021 China

**Keywords:** *Saccharomyces cerevisiae*, *atg22*, Acetic acid, Programmed cell death, Amino acid transport, Cell wall and cytomembrane

## Abstract

**Background:**

Programmed cell death (PCD) induced by acetic acid, the main by-product released during cellulosic hydrolysis, cast a cloud over lignocellulosic biofuel fermented by *Saccharomyces cerevisiae* and became a burning problem. Atg22p, an ignored integral membrane protein located in vacuole belongs to autophagy-related genes family; prior study recently reported that it is required for autophagic degradation and efflux of amino acids from vacuole to cytoplasm. It may alleviate the intracellular starvation of nutrition caused by Ac and increase cell tolerance. Therefore, we investigate the role of *atg22* in cell death process induced by Ac in which attempt is made to discover new perspectives for better understanding of the mechanisms behind tolerance and more robust industrial strain construction.

**Results:**

In this study, we compared cell growth, physiological changes in the absence and presence of Atg22p under Ac exposure conditions. It is observed that disruption and overexpression of Atg22p delays and enhances acetic acid-induced PCD, respectively. The deletion of Atg22p in *S. cerevisiae* maintains cell wall integrity, and protects cytomembrane integrity, fluidity and permeability upon Ac stress by changing cytomembrane phospholipids, sterols and fatty acids. More interestingly, *atg22* deletion increases intracellular amino acids to aid yeast cells for tackling amino acid starvation and intracellular acidification. Further, *atg22* deletion upregulates series of stress response genes expression such as heat shock protein family, cell wall integrity and autophagy.

**Conclusions:**

The findings show that Atg22p possessed the new function related to cell resistance to Ac. This may help us have a deeper understanding of PCD induced by Ac and provide a new strategy to improve Ac resistance in designing industrial yeast strains for bioethanol production during lignocellulosic biofuel fermentation.

## Background

Growing exhaustion of fossil fuel and increasing deterioration of environment shine a spotlight on cellulosic ethanol which is regarded as the most promising substitute of petrochemical resources for their abundant, available, low-cost feedstocks from forestry residues, agricultural residues and energy crops [1, 2]. However, toxic compounds such as weak acid, furans, phenolic compounds, and hydroxymethylfurfural (HMF), which were produced during lignocellulose-based saccharification and fermentation process, inhibited cell growth of *Saccharomyces cerevisiae*, the optimal microorganism of bioethanol manufacture, and declined bioethanol productivity [3]. Among all threatening inhibitors, acetic acid (Ac), the most abundant and harmful by-product in lignocellulosic hydrolysates, can inhibit cell growth and decrease alcohol productivity by disrupting cell metabolism such as intracellular acidification [4], oxidative stress and ATP depletion, inhibiting glycolytic process, suppressing amino acids uptake, impairing plasma membrane stability and selective permeability, decreasing cell wall integrity and organization, and degrading mitochondrion. Ultimately, Ac induced programmed cell death (PCD) of *Saccharomyces cerevisiae* [5, 6]. To increase Ac tolerance in yeast cells, numerous works including overexpression or deletion of single gene, manipulation of Haa1-Regulon, evolutionary engineering and genome shuffling, transcriptome remodeling and supplementation of growth media with cations were explored and delightful results were achieved [4, 7–9]. We also have shown that numerous amino acid permeases, transporters and critical proteins responsible for uptake and synthesis of amino acids are transcriptionally repressed by Ac using a RNA-Seq-based analysis and evidences from previous study showed Ac can inhibits the uptake of histidine, lysine, uracil, tryptophan, glucose, and phosphate [5, 6, 10–13]. Nonetheless, further in-depth research is indispensable for understanding the mechanisms of stress tolerance, and implementing efficient and economical strategies that used *S. cerevisiae* as microbial factories to fabricate bioethanol.

In *S. cerevisiae*, the maximal tolerance to Ac is dependent on a persistent and efficient capacity for acquiring available amino acids to maintain a basic physiological function [10, 14]. Accordingly, modulation of amino acid metabolic pools likely contributes to improve the survivability of *S. cerevisiae* upon Ac treatment. Atg22p, an obscure member of autophagy-related genes (Atg) family, is localized on the vacuolar membrane, and consisted of 528 amino acids which constitute 12 transmembrane helices with limited homologies to permeases [15]. Compared to other well-known autophagy-related genes such as *atg1*, *atg8* or *atg5*, *atg22* was unnecessary for autophagy and paid little attention to. Initially, it was deemed that *atg22* plays a vital role in cooperating with *aut4* during the last step of autophagy—autophagic bodies breaking down within lysosome/vacuole, for the slight accumulation of autophagic bodies emerged inside the vacuole because *Atg22*∆/*Aut4*∆ mutant cells under starving situation. However, further study revealed this function was not direct or crucial. *Atg22*∆ may delay autophagic bodies breakdown kinetically rather than lead autophagy-defective. Furthermore, *atg22* is proposed to be related with amino acids recycled from vacuole to cytosol after autophagic bodies degradation, that is a critical part of autophagy performing its function as an internal nutrient pool to maintain the indispensable metabolism of cells and make them survive in extreme conditions. Specifically, *atg22* is more likely to act as an effluxer mediating amino acids between vacuolar and cytosol by coordinating with another two-membrane proteins—*avt3* and *avt4*, which have similar structure and identical location to *atg22*. Previous studies indicate that *Atg22∆* can damage the uptake ability of several amino acids such as lysine, histidine and arginine. Though direct evidences of *atg22* acting as transporter of amino acid on vacuolar have not yet obtained, there is no doubt that Atg22p should go hand in hand with amino acid metabolism while it is never associated with Ac tolerance. These findings suggest new insights into how Atg22p regulates yeast cells response to Ac stress, and contributes to the exploration of the engineered *S. cerevisiae* strains with high inhibitors tolerance.

In this work, the phenotypic characterization of PCD upon Ac treatment was firstly compared between the *Atg22*Δ mutant and wild-type BY4742. The effect of overexpression of *atg22* gene on PCD under Ac stress was evaluated. Subsequently, the external and internal structure of *S. cerevisiae* mutant was observed by scanning and transmission electronmicroscopies. Further, compositions of cell wall and cytomembrane as well as the profiles of intracellular and vacuolar amino acids in *S. cerevisiae* cells were comparatively analyzed. Finally, reverse transcription quantitative real-time PCR (RT-qPCR) was employed to investigate the transcriptional regulation of stress responses and cellular metabolism by *atg22* disruption upon Ac treatment.

## Results

### *Atg22* deletion has a pro-survival role during acetic acid treatment

**Fig. 1:**
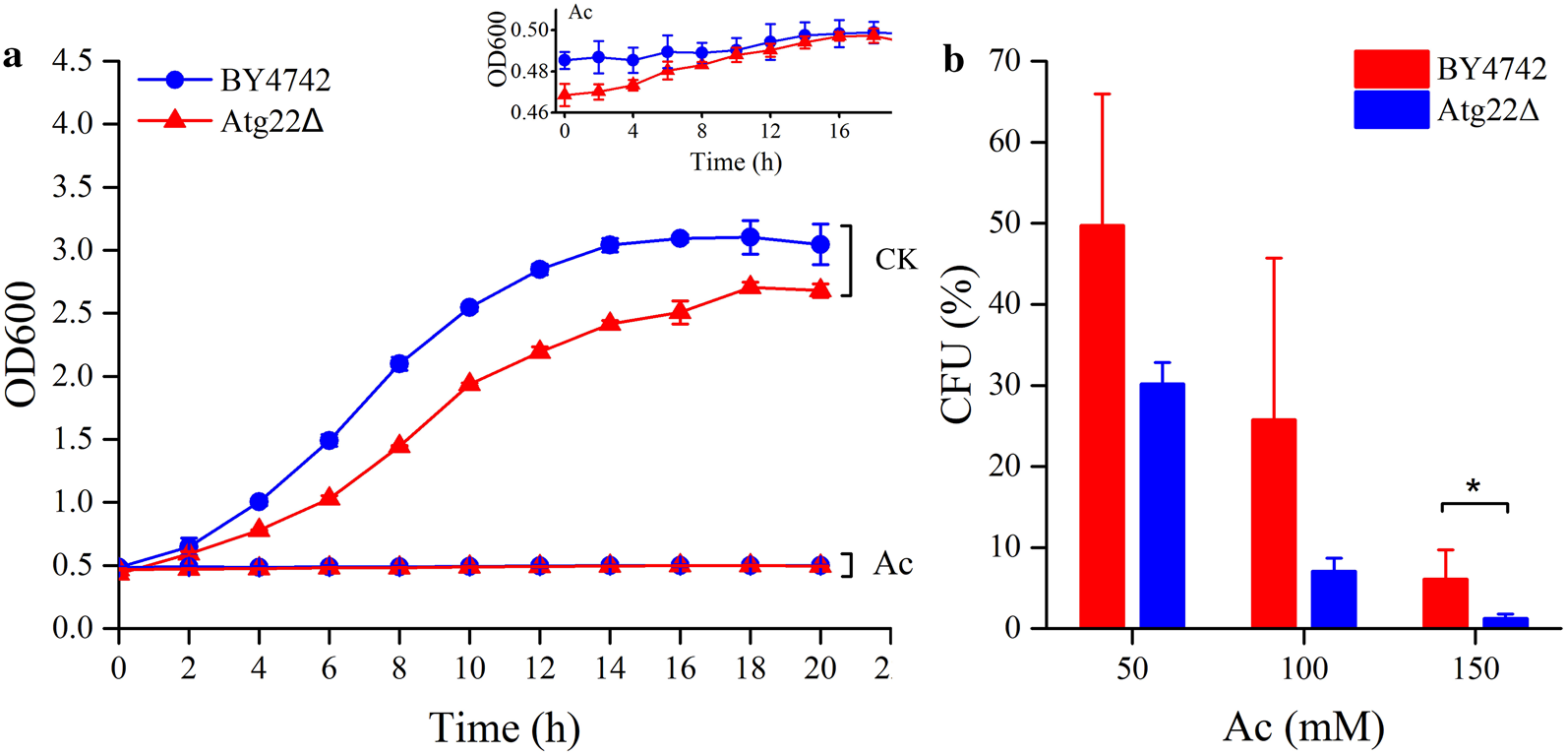
Growth curves of *S. cerevisiae* BY4742 and *Atg22*Δ (**a**), and cell survival of both strains after acetic acid treatment (**b**). The strains were cultured in SC media (pH 3.0) with acetic acid (Ac) or without 150 mM Ac (CK), and cultures at different times were assayed by optical density at 600 nm (OD_600_) (**a**). An enlarged image of growth curve during acetic acid treatment is illustrated in the insert. Viability of yeast cells treated with different concentration of acetic acid for 2 h were determined by assaying colony-forming units (CFU) after 3 days cultivation on YPD agar plates (**b**). The data represent mean ± SD (*n* = 3; *P < 0.05)

In order to assess the effects of acetic acid on cell growth and viability, the growth curves were obtained by measuring OD_600_, and cell viability was tested by counting colony-forming units. We observed that both the wild-type (WT) and *Atg22*Δ mutant were significantly growth-arrested by acetic acid stress (Fig. 1a), in accordance with the previous research [16]. The mutant showed a slower cell growth than WT in synthetic complete medium (SC) without acetic acid (CK), but *Atg22*Δ cells seemed to be growing more quickly than BY4742 until about 10 h after acetic acid (Ac) treatment, which was presented in the insert picture. Compared with the WT strain, there was an increase in cell survival of *Atg22*Δ after treatment with different concentrations of Ac (Fig. 1b). These findings indicated that the deletion of *atg22* had a pro-survival role under acetic acid stress.

### *Atg22* deletion results in inhibition of acetic acid-induced cell death

**Fig. 2:**
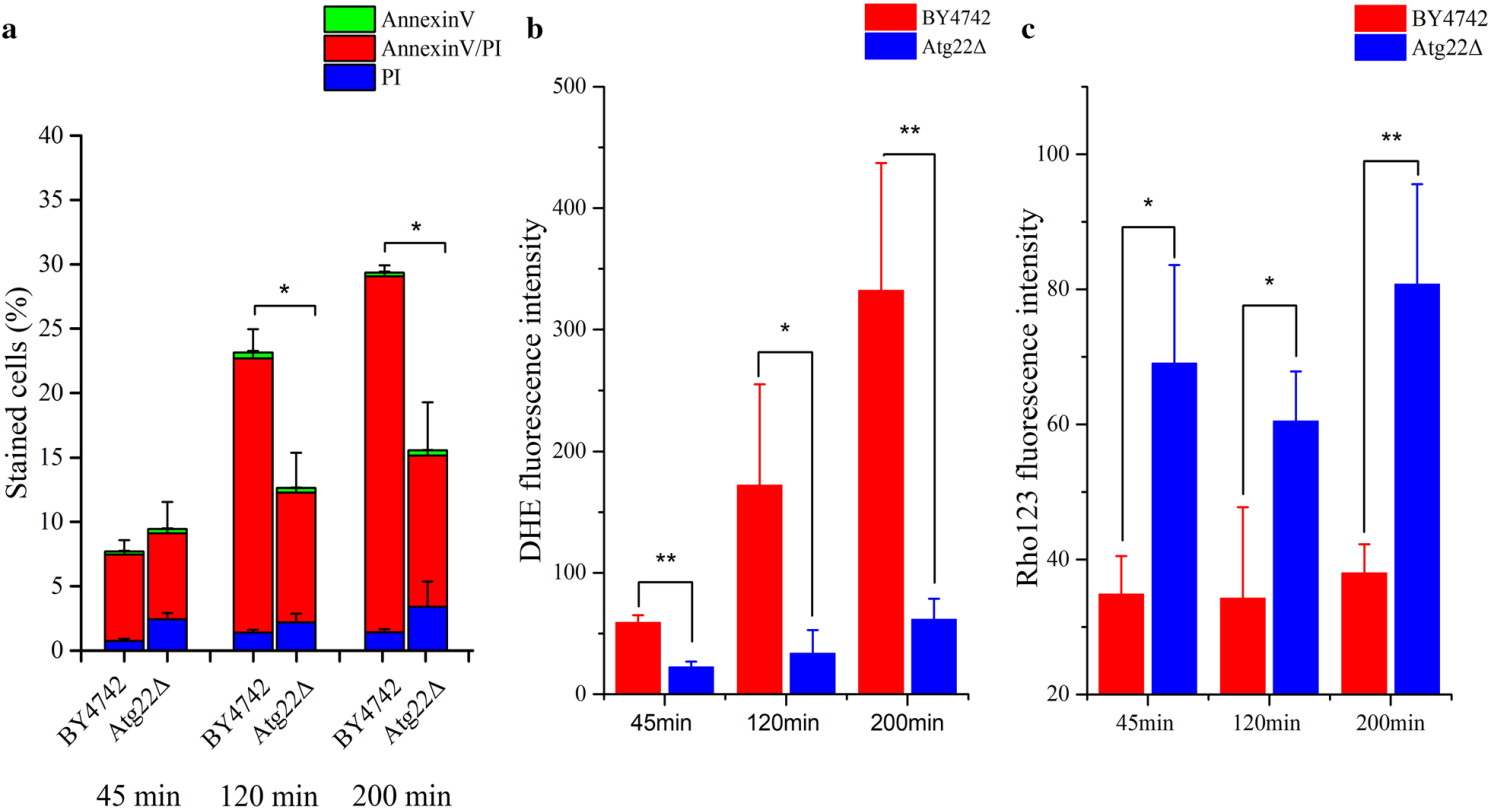
Effect of *atg22* deletion on programmed cell death induced by Ac. **a** Phosphatidylserine externalization and loss of membrane integrity were determined using Annexin V/PI costaining in BY4742 and *Atg22*Δ cells after Ac treatment. **b** ROS production of BY4742 and Atg22Δ cells was determined using DHE staining after Ac treatment. **c** Mitochondrial membrane potential was detected by Rho123 stain after Ac treatment. The data represent mean ± SD (*n* = 3; *P < 0.05, **P < 0.01). In each experiment, 10,000 cells were evaluated by flow cytometry after incubation with 150 mM Ac (pH 3.0) for 45 min, 120 min and 200 min, respectively

Yeast cells undergoing cell death induced by Ac exhibit specific markers of apoptosis [17]. In order to elucidate the role of Atg22p in cell apoptotic process induced by Ac, several apoptotic markers were analyzed for *Atg22*Δ and BY4742 cells under Ac treatment. We first assessed externalization of phosphatidylserine in the plasma membrane, a hallmark of apoptotic cells, which was assayed by a fluorescent conjugate of Annexin V-FITC. Simultaneously, we evaluated disruption of membrane integrity, a typical feature of necrosis and late apoptosis, by monitoring red fluorescence stained with propidium iodide (PI). Annexin V/PI costaining showed that Ac induced a continuous rise in exposure to phosphatidylserine and loss of plasma membrane integrity in both the control and mutant strains (Fig. 2a). The deletion of *atg22* markedly reduced Ac-induced PCD as compared to the control after 120 and 200 min treatment. Obviously, yeast cells mainly show a late apoptosis-like phenotype under the designed condition at the stress of high Ac. Deletion of *atg22* would reduce the sensitivity to Ac stress in *S. cerevisiae*. The falling mortality was accompanied by a rapid decline in the production of reactive oxygen species (ROS) (Fig. 2b) and striking increase in mitochondrial membrane potential at different times (Fig. 2c).

### Overexpression of Atg22p enhances cell death induced by Ac

**Fig. 3:**
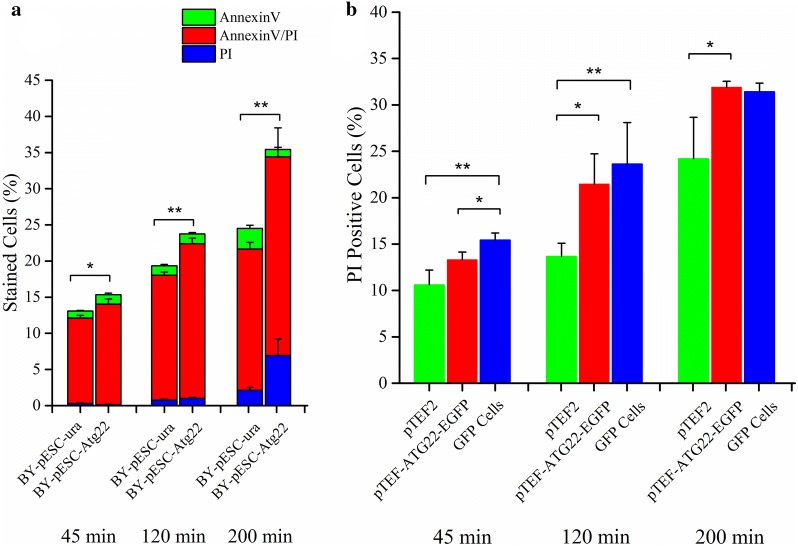
Effect of *atg22* overexpression on programmed cell death induced by acetic acid. **a** Phosphatidylserine externalization and loss of membrane integrity were determined using Annexin V/PI costaining in BY4742 cells harboring pESC-ura (BY-pESC-ura) or pESC-Atg22 (BY-pESC-Atg22) after acetic acid treatment. **b** Loss of membrane integrity was determined using PI staining in BY4742 cells harboring pTEF2 or pTEF-ATG22-EGFP after acetic acid treatment. The data represent mean ± SD (*n* = 3; *P < 0.05, **P < 0.01). Cells were incubated with 150 mM acetic acid for 45 min, 120 min and 200 min, respectively. In each experiment, 10,000 cells were then evaluated by flow cytometry. GFP cells represent the BY4742 strain harboring pTEF-ATG22-EGFP with a positive Atg22p-EGFP fluorescence by flow cytometry

To assay the role of Atg22p in response to Ac, we compared the expression of *atg22* under the control of an inducible and a constitutive promoter, respectively (Additional file 1: Figure S1). The plasmid pESC-Atg22 was constructed by sequences coding a homologous recombination *atg22* with an inducible promoter GAL10. The BY4742 strain transformed with pESC-Atg22 was induced to express Atg22p in synthetic complete medium with 2% (w/v) galactose (SG) instead of d-glucose. As shown in Fig. 3a, ectopic overexpression of Atg22p significantly accelerated the late apoptosis and necrosis induced by 150 mM acetic acid, with the aggravated phenotype of phosphatidylserine externalization and loss of plasma membrane integrity. Upon Ac treatment, Atg22p overexpression significantly decreased cell viability.

Next, Atg22p was overexpressed in BY4742 cells under the control of a constitutive TEF2 promoter, and visualized by tracing a plasmid-based fusion protein consisting of EGFP fused at the C-terminus of Atg22p. The integrity of plasma membrane was firstly detected by PI staining for evaluation of cell death in yeast cells expressing Atg22p-EGFP and empty vector control (pTEF2). Consistent with the strain overexpressing Atg22p under an inducible promoter (BY-pESC-Atg22), cell death was significantly enhanced in *S. cerevisiae* cells harboring pTEF-Atg22-EGFP under Ac stress (Fig. 3b). Although pTEF-Atg22-EGFP was transformed into BY4742, not all cells were positive for GFP. The percentage of dead cells in GFP-positive cells was markedly higher than control cells transformed with the empty vector during acetic acid treatment at 45 and 120 min. Interestingly, the former was also significantly higher than the whole cells carrying the plasmid pTEF-Atg22-EGFP at 45 min. It was clear that overexpression of Atg22p impaired the survival of yeast cells in response to Ac.

**Fig. 4:**
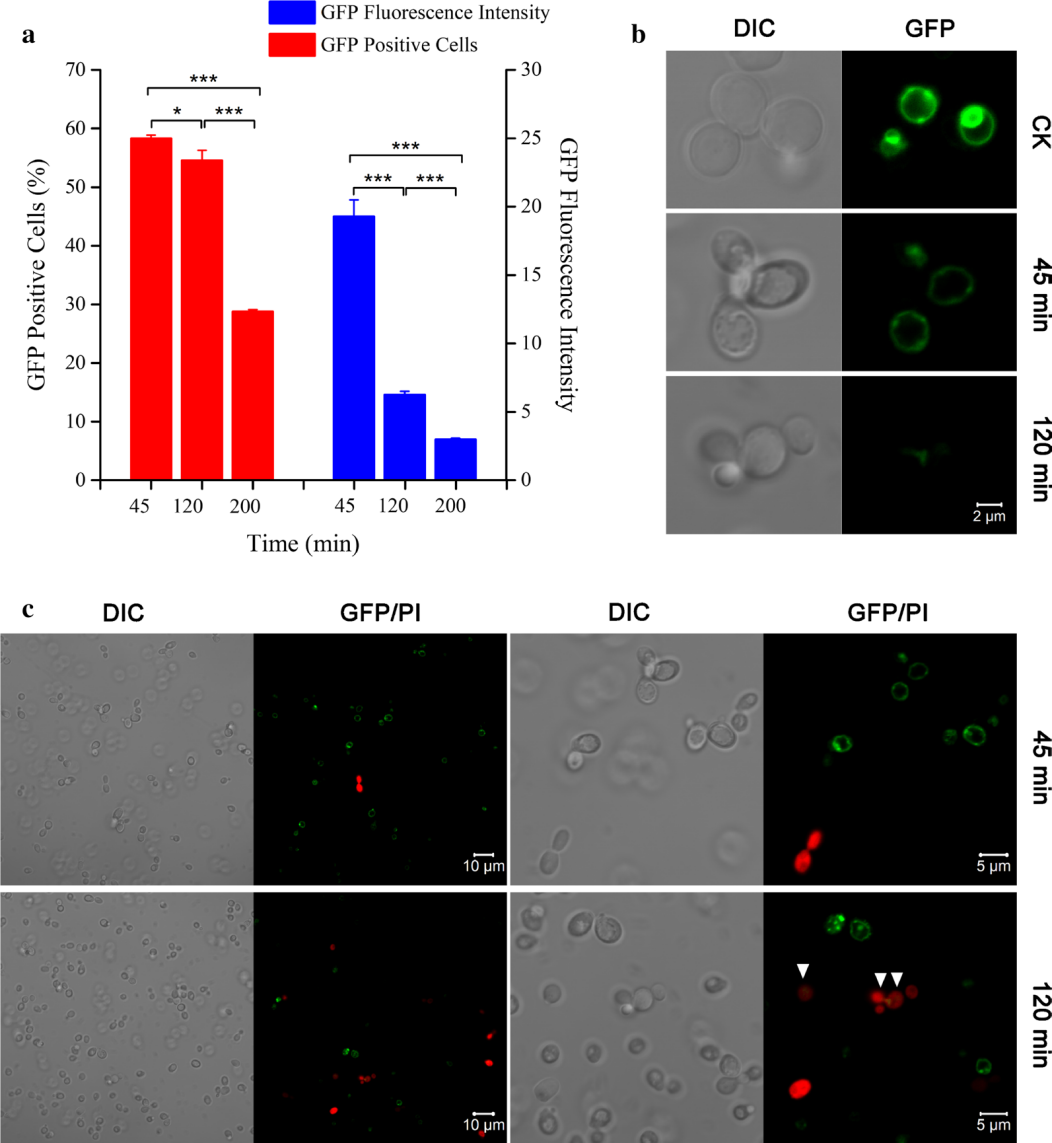
Quantification and visualization of Atg22p in yeast cells upon acetic acid stress. **a** Percentage of GFP-positive cells and mean fluorescence intensity were measured by flow cytometry in BY4742 cells harboring pTEF-Atg22-EGFP. **b** Representative images of differential interference contrast (DIC, left panels) and green fluorescence microscopy (GFP, right panels) were obtained from cells harboring pTEF-ATG22-EGFP before (CK) and after exposure to 150 mM acetic acid for 45 min and 120 min, respectively. **c** Representative DIC (first and third panels) and GFP/PI images (second and fourth panels) were obtained from cells harboring pTEF-ATG22-EGFP treated with 150 mM acetic acid for 45 min and 120 min, respectively. The GFP/PI images were merged with PI staining and GFP fluorescence, and GFP-positive cells with PI staining were indicated by white arrows. Data represent mean ± SD (*n* = 3; *P < 0.05, **P < 0.01, ***P < 0.001). In each experiment, 10,000 cells were evaluated by flow cytometry after incubation with 150 mM acetic acid for 45 min, 120 min and 200 min, respectively

The GFP-positive cells and mean fluorescence intensity (MFI) fell dramatically in all cells harboring pTEF-Atg22-EGFP in the presence of Ac (Fig. 4a). Figure 4b shows that Atg22p mainly located in the vacuolar membrane. A high fluorescence intensity was observed in cells cultured in SC medium without Ac treatment. Confocal microscopy images confirmed that MFI was significantly decreased upon Ac stress in cells expressing Atg22-EGFP fusion protein. Simultaneously, there was a significant reduction of GFP-positive cells after Ac treatment (Fig. 4c), which was consistent with the above data measured by flow cytometry. Interestingly, the GFP-positive cells showed a higher cell death rate (white arrow) than the whole cells treated with acetic acid for 120 min. Taken together, the levels of Atg22 may determine the sensitivity of yeast cells in response to Ac-induced apoptosis.

### *Atg22* changes cell morphology under Ac treatment and regulates cell wall composition

**Fig. 5:**
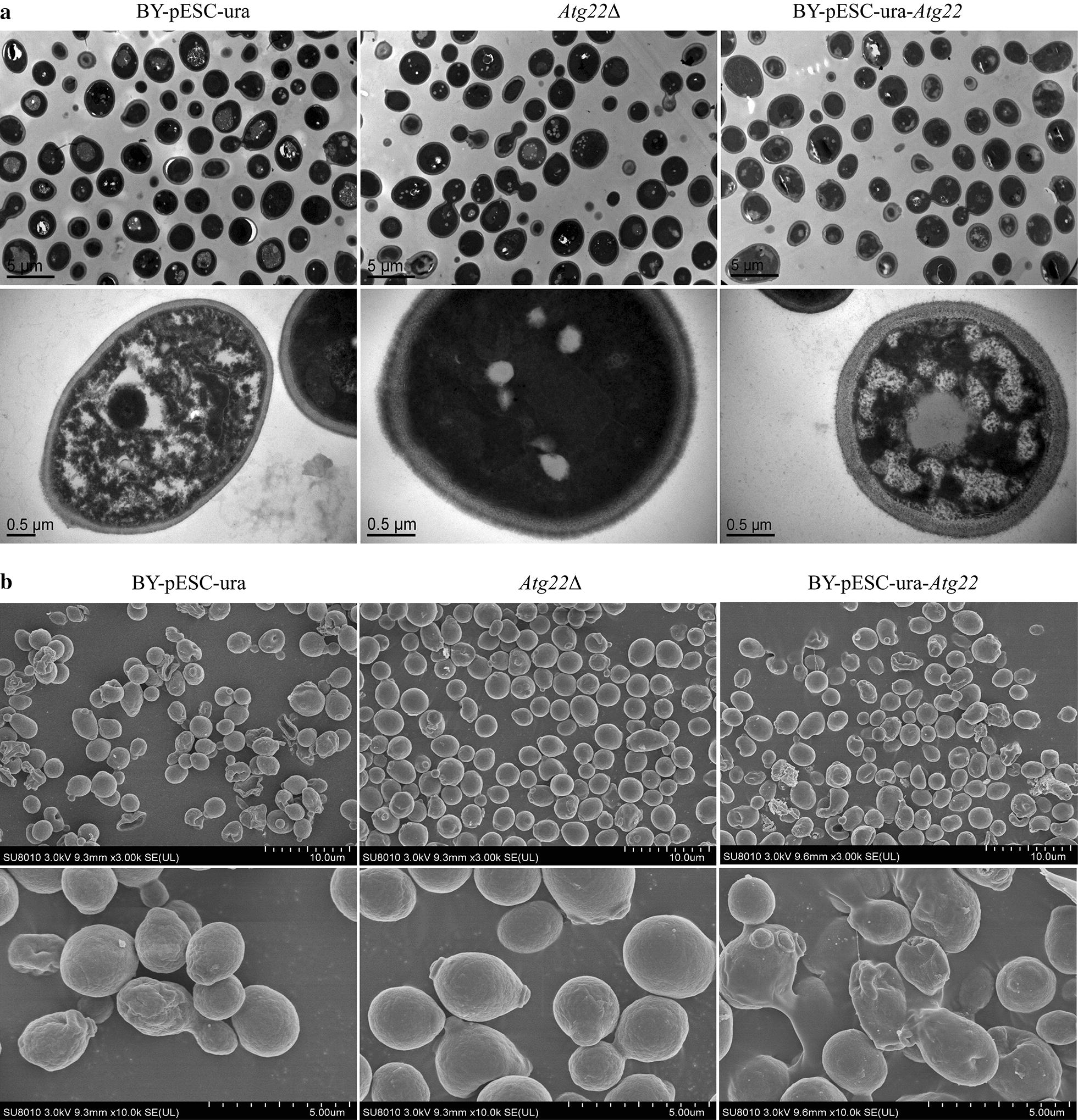
Morphology changes under 150 Mm Ac for 120 min treatment. **a** Interior structure changes observed by TEM. **b** Surface structure changes detected by SEM. All cells exposed to 150 Mm Ac for 120 min, and every and biological triplicate were performed

**Fig. 6:**
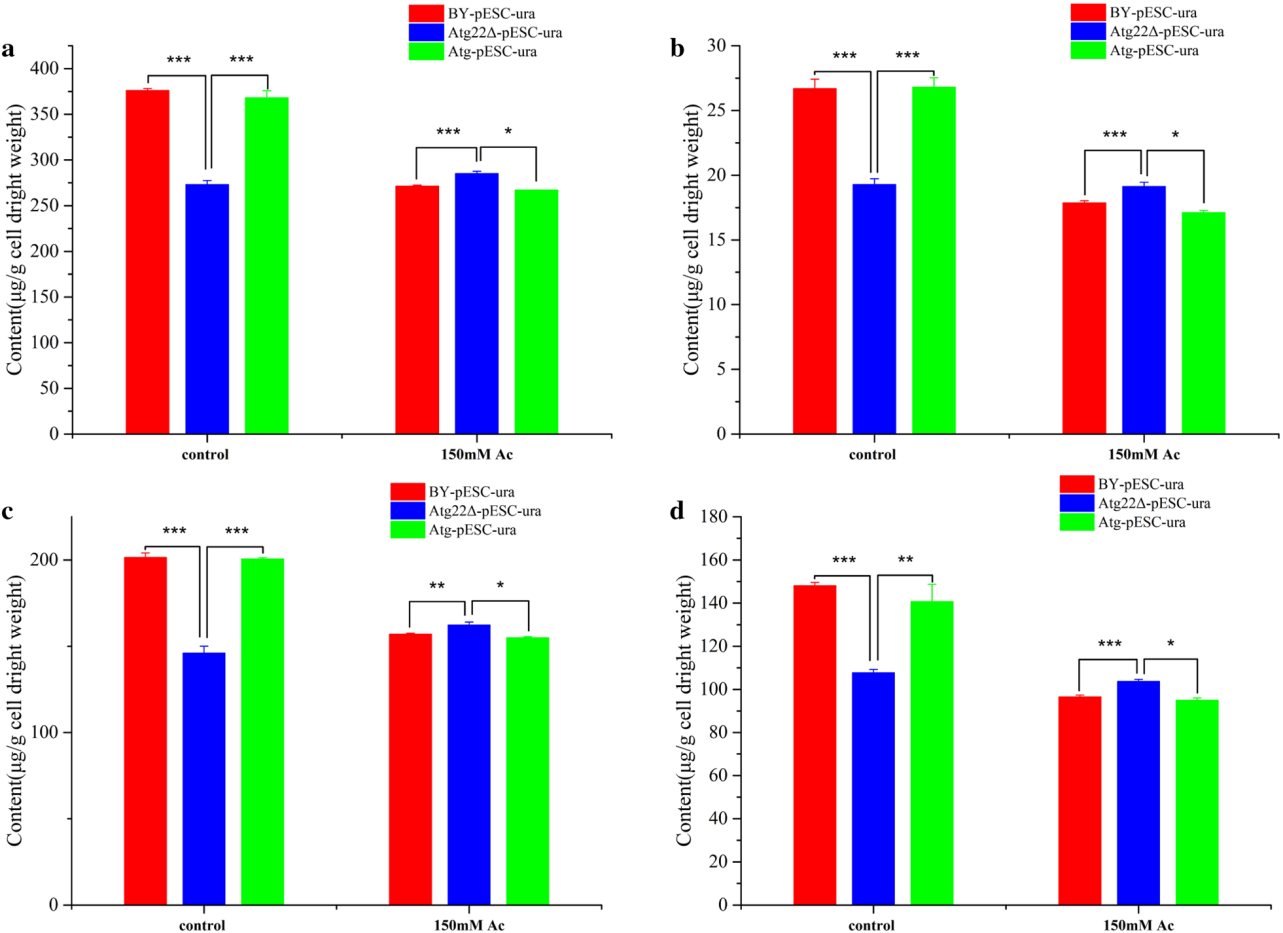
Atg22p affects cell wall compositions in the control and Ac exposure conditions. **a** Total polysaccharide, **b** chitin content, **c** glucan content, **d** mannan content. Data represent mean ± SD and error bars determined by the *t* test (*n* = 3; *P < 0.05, **P < 0.01, ***P < 0.001)

Cell wall generally signaled outer environmental stressors. To understand the role of *atg22* affecting the change of cell wall under acetic acid, we adopted scanning electron microscopy (SEM) and transmission electron microscopy (TEM) to observe the surface and interior structure of *S. cerevisiae* under Ac and without treatment, respectively. In our previous study, Ac erosion made cells vulnerable and weak by thinning cell wall, abolishing lipid droplets accumulation, disintegrating organelles and increasing cytoplasm vacuolation. We compared the cell status of BY4742-pESC-ura, Atg22Δ-pESC-ura and BY4742-pESC-Atg22. Clearly presented, more fragments and wrinkles were observed on the surface of BY4741 and *Atg22* overexpression strains (Fig. 5a). What’s more, TEM observation reveals *atg22* existence or overexpression may exacerbate the destruction of interior structure significantly. Additionally, the breakdown in *atg22* deletion strain was partly compared with the extensive and exhaustive collapsing emerged in BY4742-pESC-ura and BY4742-pESC-Atg22 (Fig. 5b). To explore the reason behind this phenomenon, we analyzed cell wall components. The content of total polysaccharide is 37.8% lower under control situation as compared to BY4742-pESC-ura, while 5% higher after 150 mM Ac exposure. Moreover, Ac treatment decreased 38.6% and 37.8% of total polysaccharide in control strain and overexpression strain restrictively, but had no influence on *Atg22*Δ strain (Fig. 6a). In the control group, *atg22* deletion resulted in the loss of amounts of glucan, mannan and chitin by 27.5%, 37.4% and 27.7%, respectively. Upon 150 mM Ac treatment, those constituents decreased by 22%, 34.8%, 32.8% in BY4742-pESC-ura and 22.7%, 32.4%, 36.1% in BY4742-pESC-Atg22, respectively, in contrast to the control. Interestingly, glucan was the only one of polysaccharides upregulated in Atg22Δ, indicating *atg22* affects cell wall compositions synthesis (Fig. 6b–d).

### Atg22 regulates plasma membrane rigidity and compositions

**Fig. 7:**
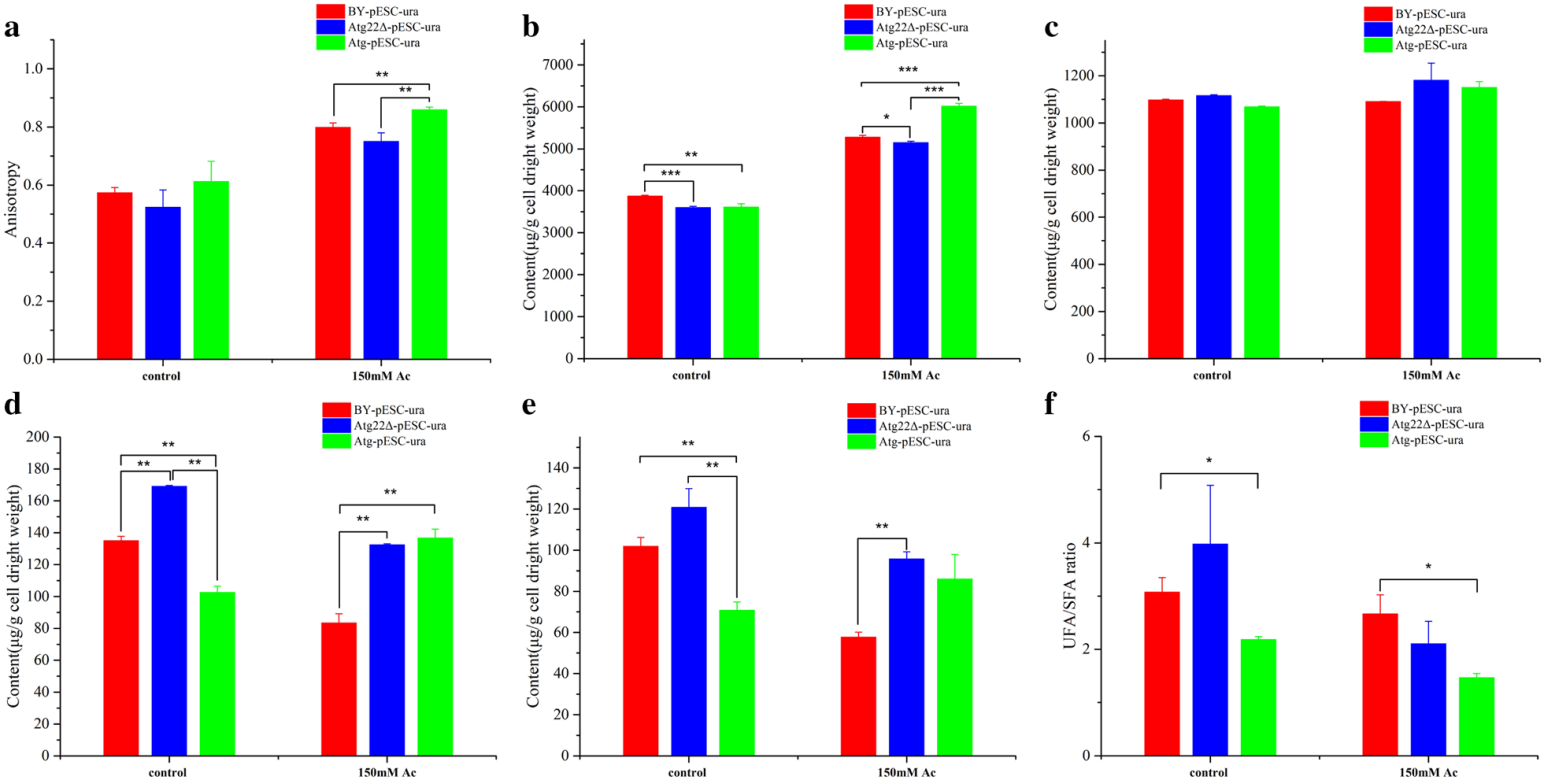
Effect of *atg22* on membrane rigidity and total compositions. **a** Assays of membrane rigidity, **b** total phospholipid, **c** total sterol, **d** total fatty acid, **e** total UFA, **f** UFA/SFA. All experiments were implemented in biological triplicate. Data represent mean ± SD and error bars determined by the *t* test (*n* = 3; *P < 0.05, **P < 0.01, ***P < 0.001)

**Fig. 8:**
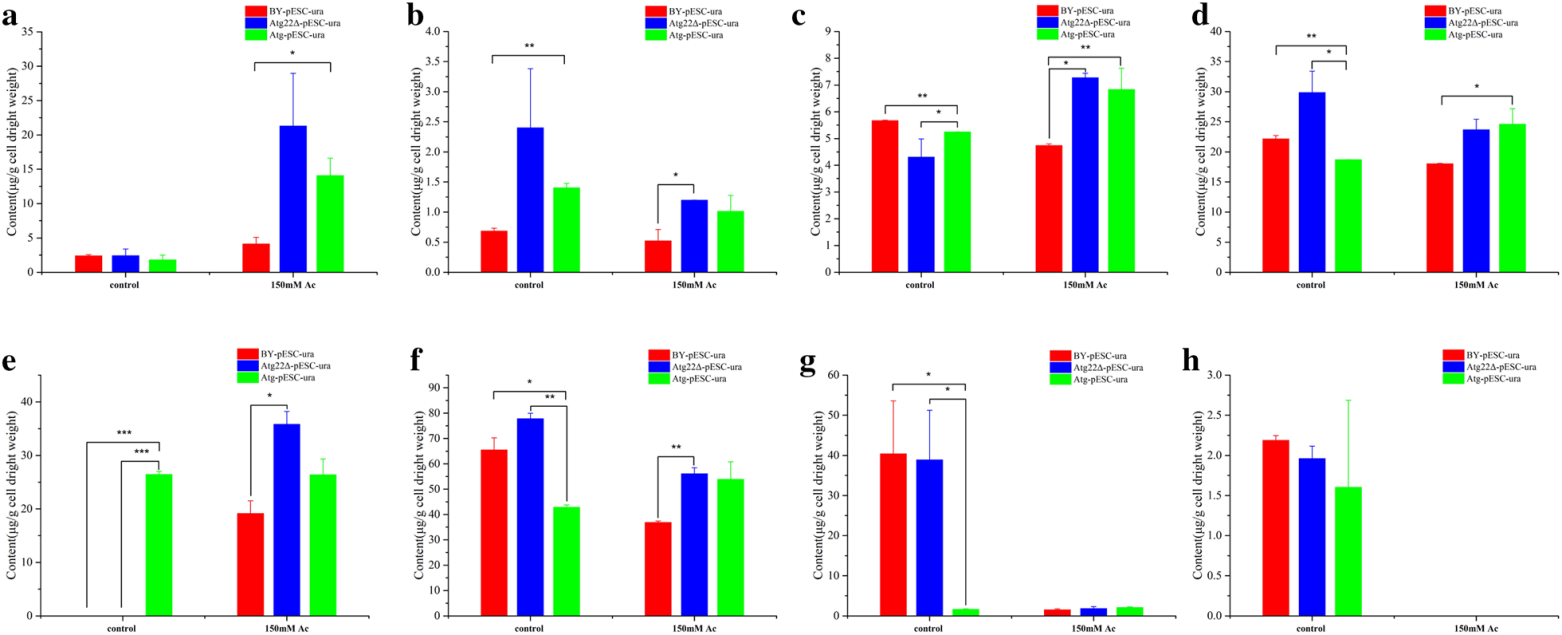
Effect of *atg22* on fatty acid content of the plasma membrane. **a** Lignoceric acid content, **b** myristic acid content, **c** stearic acid content, **d** palmitic acid, **e** elaidic acid content, **f** palmitoleic acid content, **g** oleic acid content, **h** linolelaidic acid content. Data represent mean ± SD and error bars determined by the *t* test (*n* = 3; *P < 0.05, **P < 0.01, ***P < 0.001)

**Fig. 9:**
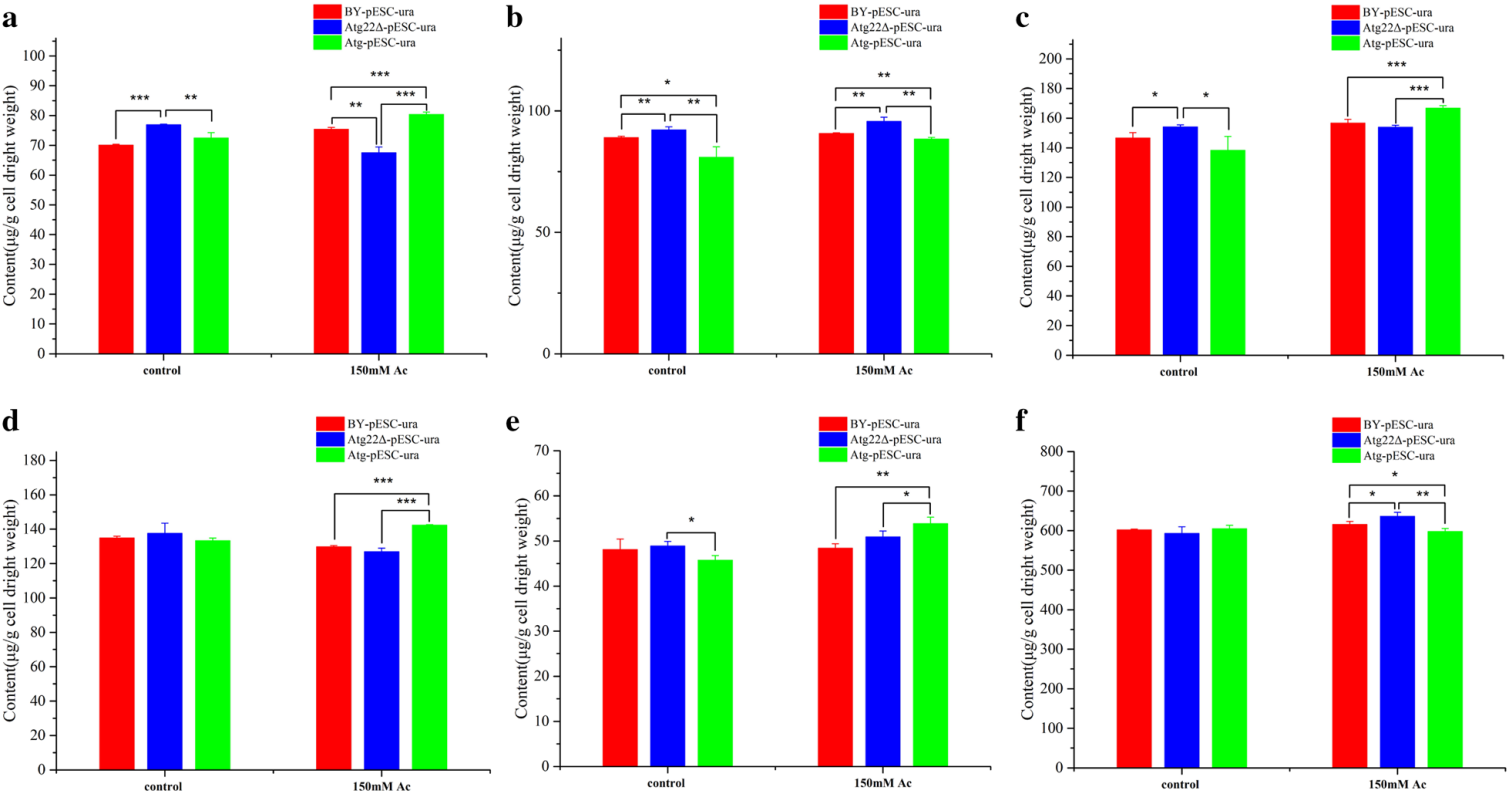
Influence of *atg22* on sterol content of the plasma membrane. **a** Squalene content, **b** lanosterol content, **c** 4,4-dimethylzymosterol content, **d** zymosterol content, **e** fecosterol content, **f** ergosterol content. Data represent mean ± SD and error bars determined by the *t* test (*n* = 3; *P < 0.05, **P < 0.01, ***P < 0.001)

Fluorescence polarization results revealed that cell membrane rigidity was 8.7% higher in the *Atg22*Δ strain than in the wild-type one but that the level in the *atg22* overexpression strain was similar to the wild-type strain without Ac treatment. However, Ac caused cell membrane rigidity to decrease by 39.2%, 40.4%, 43.2% in BY4742-pESC-ura, Atg22Δ-pESC-ura and BY4742-pESC-Atg22, respectively. However, cell membrane rigidity in *atg22* deletion strain showed 6% and 12.7% higher than the control and the overexpression strain, respectively (Fig. 7a). We compared the membrane components among three strains. Upon Ac exposure, the total content of fatty acid, saturated fatty acids, and unsaturated fatty acids decreased by 39%, 20% and 44% in BY4742-pESC-ura; reduced 22%, increased 20%, reduced 21% in *Atg22*Δ strain; upregulated 33%, 80%, 21% in overexpression strain. Under control conditions, total content of fatty acid in *Atg22*Δ was 25% and 64% higher than control and overexpression strain; total content of saturated fatty acid in *Atg22*Δ was 28% and 72% higher than control and overexpression strain; 18% and 70% higher than control and overexpression strain in total content of unsaturated fatty acid (Fig. 7d–f, Additional file 1: Table S1). The content of myristic acid, palmitic acid, stearic acid, lignoceric acid, palmitoleic acid, oleic acid, cis-4,7,10,13,16,19-docosahexaenoic acid in *Atg22*Δ is more enriched than that in wild-type and overexpression groups. The total content of fatty acid in *Atg22*Δ was 58% higher than the control strain (Fig. 7d); total content of saturated fatty acids in *Atg22*Δ 92% and 14% higher than the control and overexpression strain, respectively; 66% and 11% higher than control and overexpression strain in total content of unsaturated fatty acids (Fig. 7e, f). The content of myristic acid, palmitic acid, stearic acid, lignoceric acid, palmitoleic acid, elaidic acid, oleic acid, cis-4,7,10,13,16,19-docosahexaenoic acid in *Atg22*Δ strain is 2.87, 1.31, 1.54, 5.14, 1.52, 1.87, 1.21, 2.76-fold of BY4742-pESC-ura and 1.48, 0.96, 1.07, 1.51, 1.04, 1.36, 1.2, 1.19-fold of the overexpression strain (Fig. 8a–h). Further, membrane sterol levels were investigated to determine whether *atg22* affects sterol biosynthesis. The content of sterols was presented in Additional file 1: Table S2, and we found that the deletion of *atg22* upregulated the levels of squalene. No change was observed in *atg22* overexpression strain except 10% decrease of squalene. At 150 mM Ac, in BY4742-pESC-ura only 4,4-dimethylzymosterol increased 7% compared with the control. In *Atg22*Δ, squalene and zymosterol decreased by 12%, 8%, respectively. The findings implied that overexpression of *atg22* increased the levels of sterols except for ergosterol (Fig. 9a–f).

**Fig. 10:**
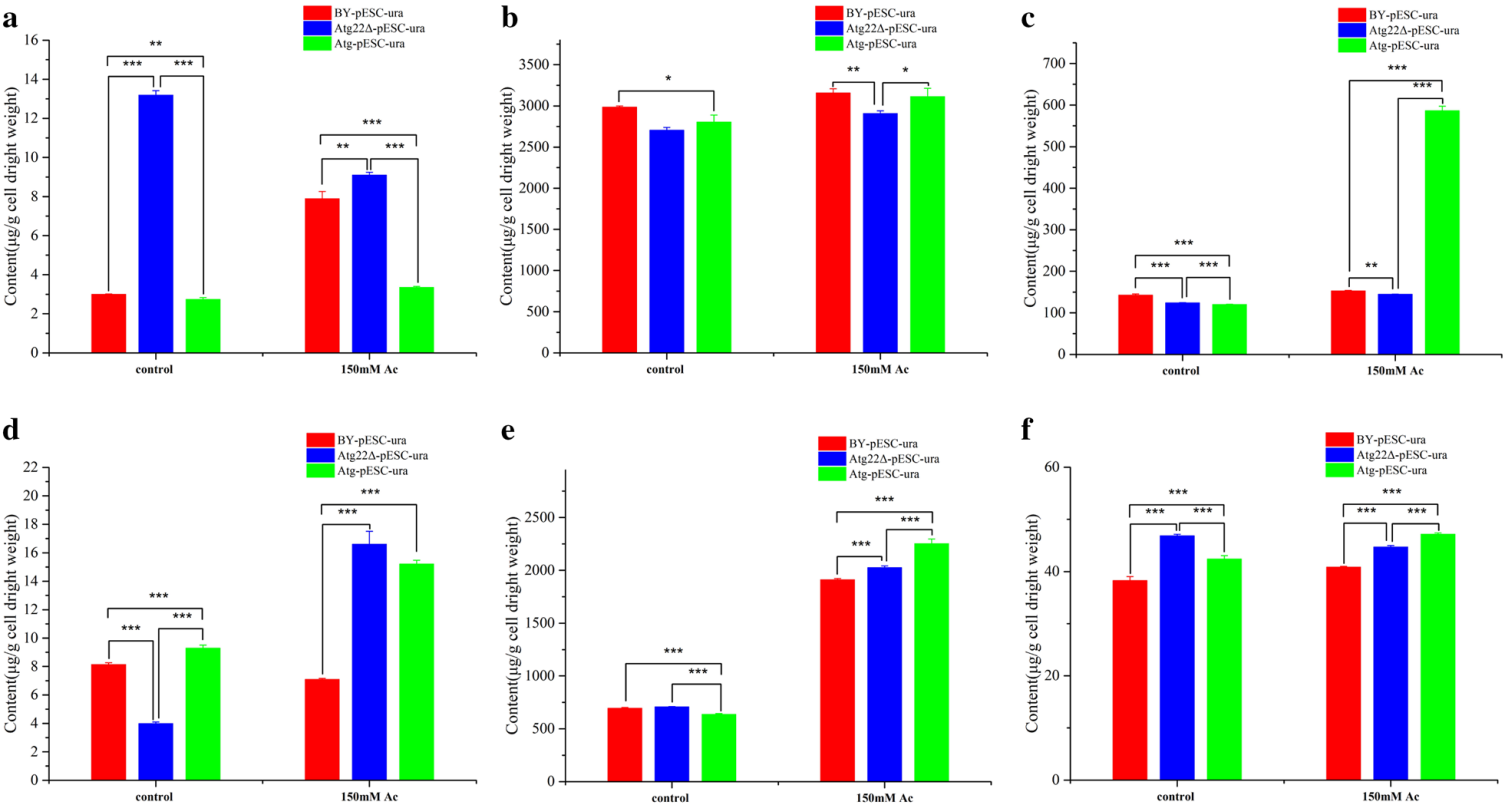
Influence of *atg22* on phospholipid content of the plasma membrane. **a** PA, **b** PC, **c** PI, **d** PG, **e** PE, **f** PS. Data represent mean ± SD and error bars determined by the *t* test (*n* = 3; *P < 0.05, **P < 0.01, ***P < 0.001)

Next, the content of phospholipids in cell membrane was comparatively analyzed (Fig. 10a–f). We found the total concentrations decreased by 7% and 6.6% in *atg*22 deletion and overexpression strains, respectively (Additional file 1: Table S3). Without Ac treatment, phosphatidic acid (PA), phosphatidylserine (PS) increased by 341%, 22% and phosphatidylglycerol (PG), phosphocholine (PC), phosphatidylinositol (PI) decreased by 51%, 9%, 13% in Atg22Δ strain, respectively. In *atg22* overexpression strain, PA, PC, phosphatidylethanolamine (PE), PI down by 9%, 6%, 8%, 16% and PG, PS up by 14%, 11%, respectively, compared with the control. At 150 mM Ac, the total phospholipids increased 36%, 43%, 66% in BY4742-pESC-ura, *Atg22*Δ and BY4742-pESC-Atg22, respectively. Concretely, in BY4742-pESC-ura strain PA, PE up by 164%, 175%, respectively. Meanwhile, in *Atg22*Δ strain, PG, PE increased by 317%, 186% while PA and PS decreased by 31%, 5%, respectively. In BY4742-pESC-Atg22 strain all component contents were upregulated in which PA, PG, PE, PI increased by 22%, 63%, 254%, and 388%, respectively. The present findings implied that vacuolar *atg22* has directly regulated yeast cell death under Ac.

### The *Atg22*Δ cells accumulate cytosolic amino acids during Ac treatment

**Fig. 11:**
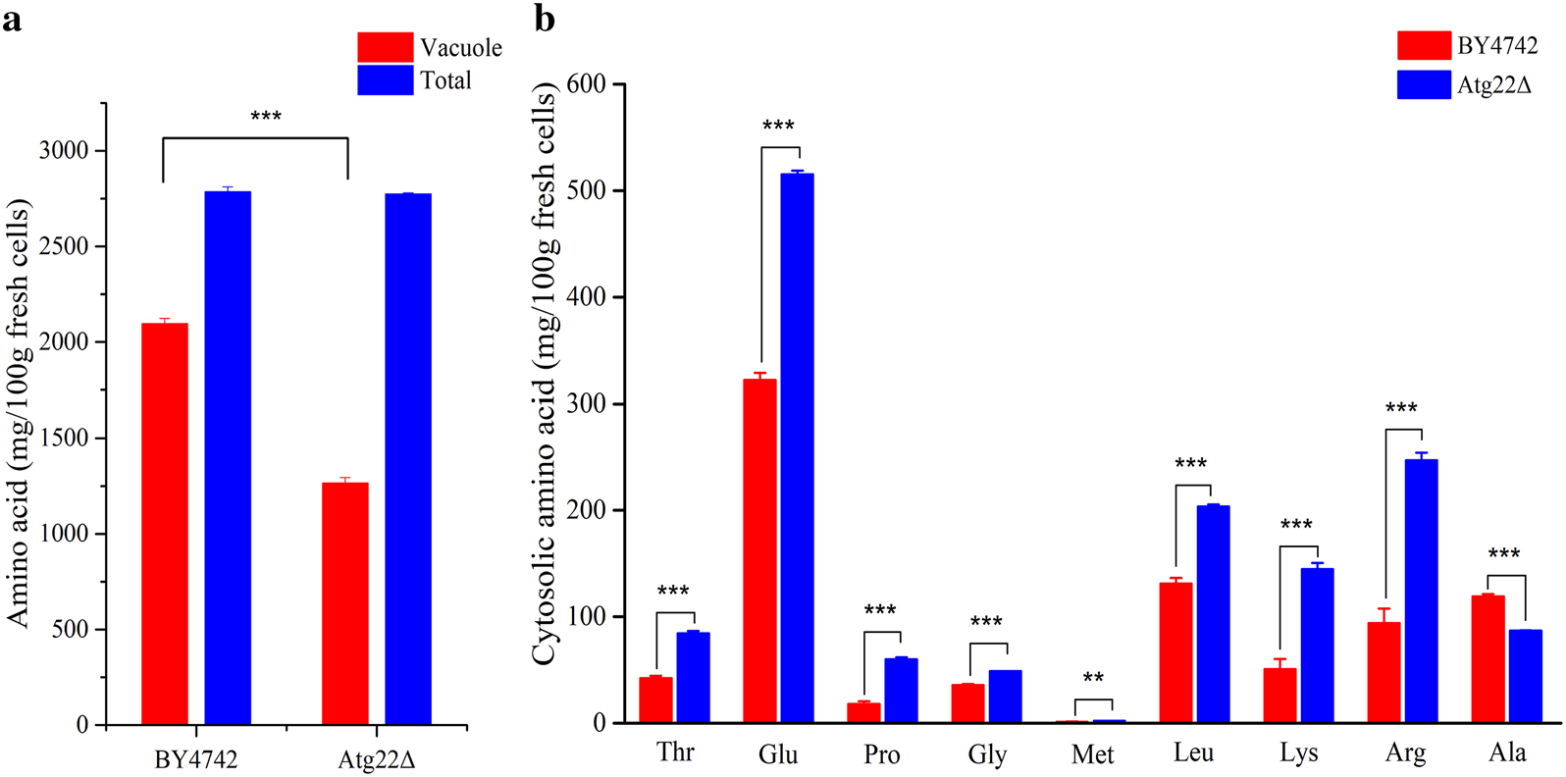
Effect of *atg22* deletion on intracellular amino acid pools during acetic acid treatment. **a** Vacuolar and total amino acid pools, **b** cytosolic concentrations of representative amino acids. The data represent three independent experiments and error bars determined by the *t* test (*P < 0.05, **P < 0.01, ***P < 0.001)

Vacuolar Atg22p is required for efflux of amino acids and breakdown of autophagic bodies [18]. The contents of total amino acids and vacuolar amino acids were measured for evaluating the effect of Atg22p deficiency on intracellular amino acid pools. As presented in Fig. 11a, wild-type BY4742 cells accumulated higher concentration of vacuolar amino acids compared with *Atg22*Δ mutant, while there was insignificant difference in total amino acids between the two strains upon acetic acid treatment. The concentration of cytosolic amino acids in mutant was 2.2-fold higher than the WT strain (*P *< 0.001). Moreover, the cytosolic contents of threonine, glutamic acid, proline, glycine, methionine, leucine, lysine, and arginine were obviously increased by *atg22* mutation (Fig. 11b). Only the content of alanine in cytosol decreased significantly. Ac was reported to induce severe nutrient limitation in yeast [5, 6, 10]. These data suggest that Atg22p probably plays a distinct role in regulation of amino acid pools between cytosol and vacuole upon Ac stress, and the deletion of *atg22* leads to cytosolic accumulation of amino acids aiding yeast cells under amino acid starvation induced by Ac.

### Deletion of *atg22* promotes gene expression in heat shock protein family, cell wall integrity pathway and autophagy in response to Ac stress

**Fig. 12:**
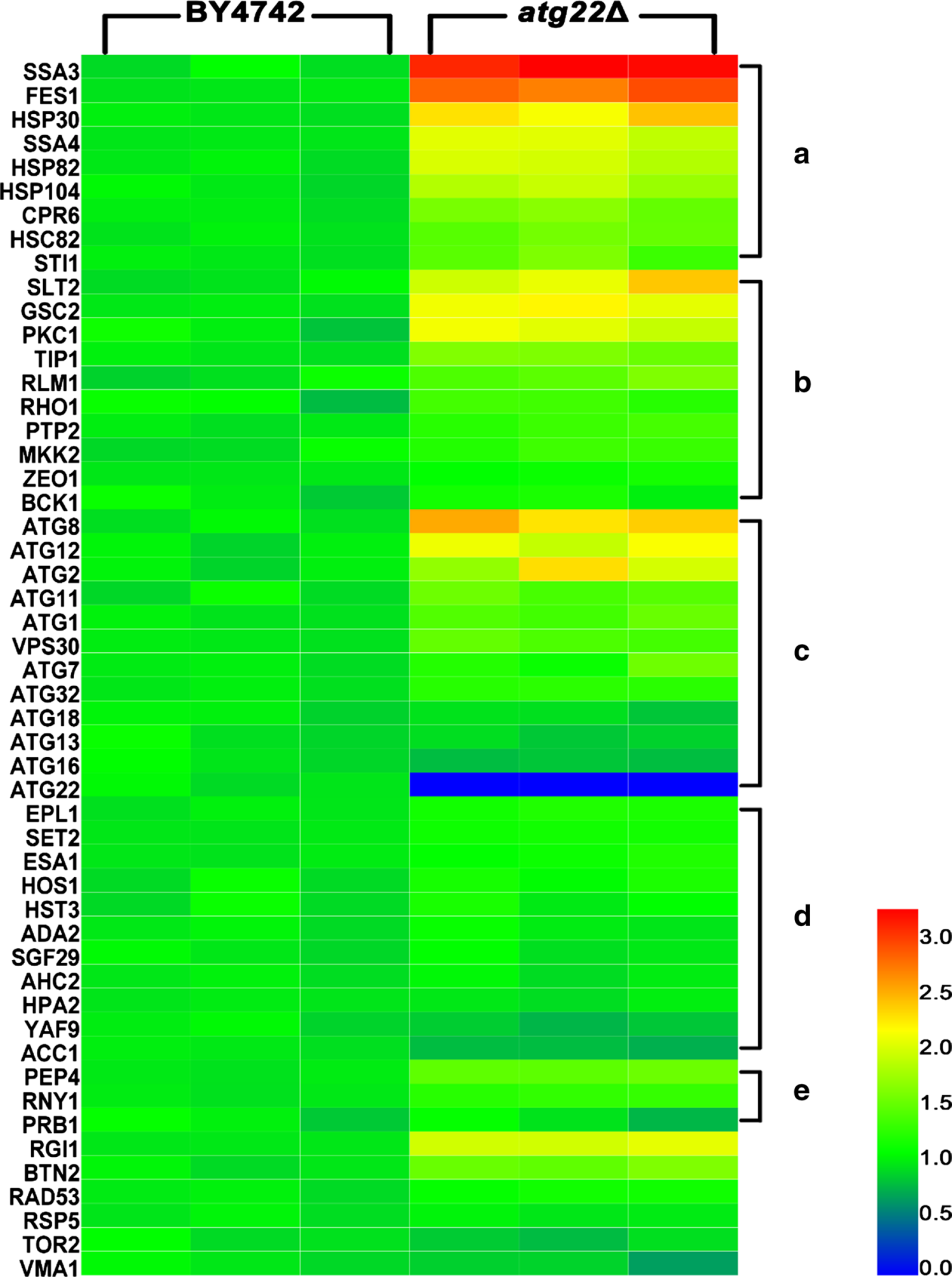
Quantitative real-time PCR (qPCR) analysis of gene expression under acetic acid stress. Gene expression of *S. cerevisiae* BY4742 and *Atg22*Δ strains was compared by fold changes after treatment with 150 mM acetic acid for 120 min. **a** Corresponding genes were categorized by functions involved in heat shock protein family, **b** cell wall integrity pathway, **c** autophagy, **d** histone acetylation and deacetylation, **e** vacuolar degradation. Expressions for each gene were presented in relative fold changes against that of BY4742 after normalization. Red indicates the enhanced expression, blue for the repressed expression, and green for no significant changes. Scales of expressions were indicated by an integrated color bar at the right

In our previous study [2] and the relevant report [19], many differentially expressed genes were identified to be involved in stress responses and Ac-induced PCD by RNA-Seq-based transcriptomic analysis. In this work, 51 key genes in related biological processes were as well selected to investigate the protective role of *atg22* deletion against cell death induced by Ac using RT-qPCR. Upon Ac stress, the mRNA abundance of 20 genes showed a significant increase in *Atg22*Δ strain as compared with the wild-type BY4742 under the same culture conditions. The upregulated genes probably contributed to improve Ac tolerance in the mutant, mainly involved with heat shock protein family (*ssa3*, *fes1*, *hsp30*, *ssa4*, *hsp82*, *hsp104*, *cpr6*, *hsc82*, *sti1*), cell wall integrity pathway (*slt2*, *gsc2*, *pkc1*, *tip1*, *rlm1*), autophagy (*atg8*, *atg12*, *atg2*), vacuolar proteolysis (*pep4*), and other processes (*rgi1*, *btn2*). Especially, the mRNA levels of all the investigated genes encoding heat shock proteins in the mutant were significantly enhanced more than 1.5-fold (*P *< 0.01) as compared with the control. However, there was insignificant change in transcription of all 11 genes involved in histone acetylation and deacetylation (Additional file 1: Table S4), indicating the cellular acetylation balance was not significantly changed by *atg22* deletion under Ac pressure. Hence, *atg22* disruption activated intensively the transcription of genes encoding stress-related proteins to improve the survival upon Ac (Fig. 12).

## Discussion

**Fig. 13:**
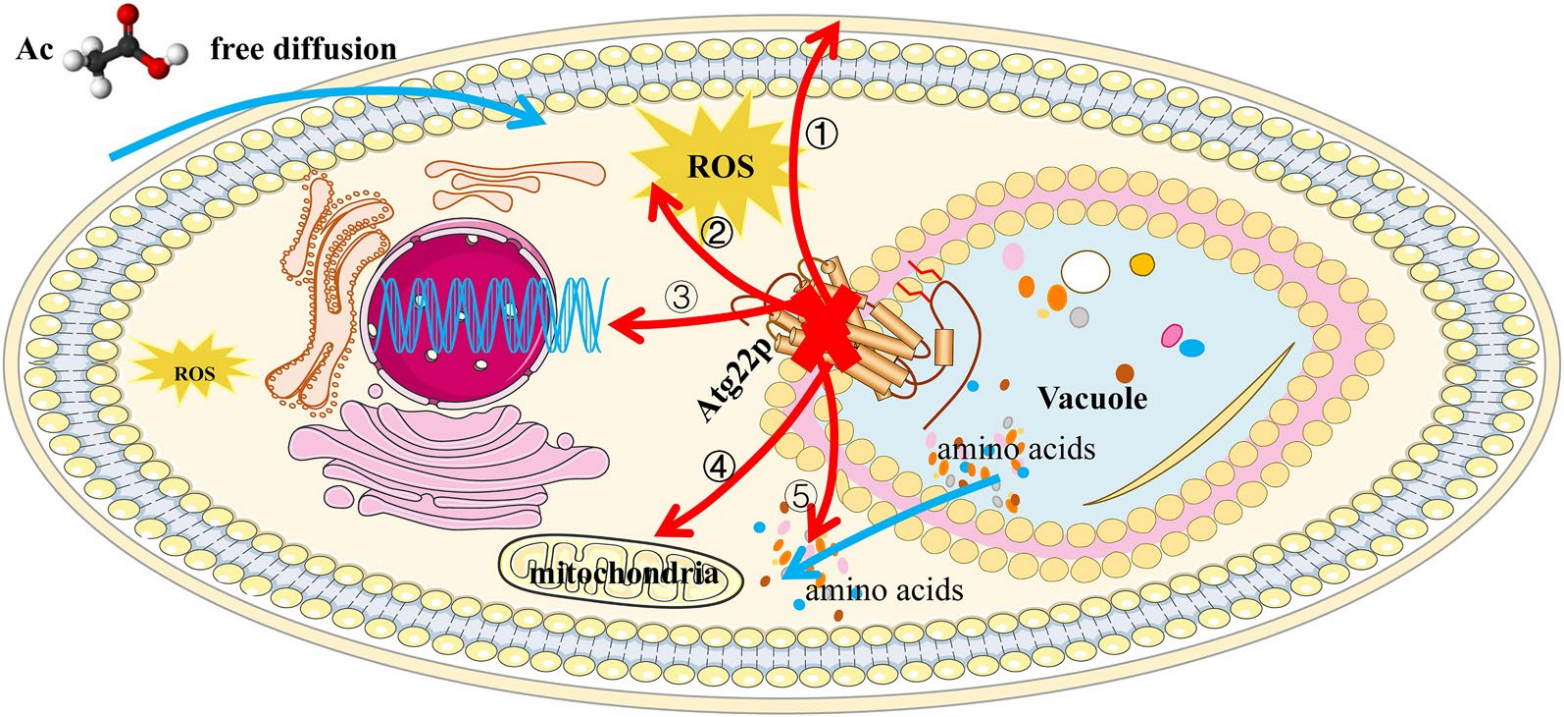
Schematic diagram of Atg22 deficiency contributes to reduce programmed cell death induced by acetic acid in *Saccharomyces cerevisiae.* ① Obstruction of Ac entrance by influencing compositions, function of cell wall and membrane. ② Decreasing accumulation of ROS in cytoplasm. ③ Upregulation of genes expression in heat shock protein family, cell wall integrity pathway and autophagy. ④ Maintaining mitochondrial membrane potential. ⑤ Changing amino acids between vacuole and cytoplasm promotes amino acids flowing from vacuole to cytoplasm

Multiple efforts have been made to improve Ac resistance and elucidated mechanisms of stress resistance in yeast [4, 20, 21]. However, little attention is paid to the relationship between Ac resistance and *atg22*, a small membrane-spanning protein on vacuole of *S. cerevisiae*. In this work, we discovered the function of *atg22* in the PCD induced by Ac through affecting cell wall synthesis, cell membrane structure and function, and amino acids transport between cytosol and vacuole, stress genes expression with regards to HSP family, Atg family and CWI, the postulated regulation pathway shown in Fig. 13. These findings emphasize the communication role of vacuolar Atg22p involved in the PCD under stress factors.

### Programmed cell death triggered

Interestingly, the *Atg22*Δ mutant presents better survivability in acetic acid-containing medium compared with WT strain and *atg22* overexpression one. By contrast, overexpression of *atg22* enhanced acetic acid-induced cell death, and the increased intracellular degradation of proteins and organelles in this process led to a sharp decline in proportion and mean fluorescence intensity of GFP-positive cells. This result was proved by the observation of Annexin V/PI showing *atg22* deletion alleviated PCD and the overexpression strain aggravated. In turn, Ac induced the degradation of Atg22p. Reactive oxygen species (ROS) accumulation and mitochondria degradation were also involved in Ac-induced apoptosis [17, 22]. ROS production during cell metabolism by mitochondrial electron transport chain or non-mitochondrial pathway is beneficial to signal transmission [23]. However, the elevated accumulation of ROS triggered by Ac cause lipid peroxidation, protein oxidation, vacuolar acidification, genetic damage and sphingolipids decrease [24, 25]. Mitochondria by means of releasing cytochrome accomplished double mission of electron donation and ROS scavenging [26]. It is exhibited that less ROS production in the cytoplasm and higher mitochondrial membrane potential (ΔΨm) were detected in *Atg22*Δ mutant. The data mean *atg22* absence might suppress ROS formation to protect the damage of mitochondria (Fig. 13). All of this attest to the fact that *Atg22*Δ showed a better performance and superior survivability in the presence of Ac. In other words, deletion of *atg22* gene in yeast contributes to reduce programmed cell death induced by acetic acid in *S. cerevisiae*.

### Cell wall compositions and integrity

In budding yeast, cell wall was supposed as dynamic organelle that played vital role in the maintenance cell morphology, mechanical strength, proliferation and the first barrier to protect cell from extracellular environment pressure [27]. Our preceding research revealed that Ac treatment significantly repressed cell wall organization particularly glucan biosynthesis at mRNA level and resulted in cell wall thinner with a darker color [2]. However, in this study we found the cell wall of *Atg22*Δ showed more integrity than BY4741 and overexpression strain after Ac treatment. Ac treatment sharply reduced cell wall composition of glucan, mannan and chitin in BY4741-pESC-ura and BY4741-pESC-Atg22 while had no influence on *Atg22*Δ. Meanwhile, disruption of *atg22* enhances transcription of key genes involved in cell wall integrity pathway (CWI pathway) (*tip1*, *pkc1*, *slt2*, *rlm1*, *gsc2*) which may provide protection to cells against acetic acid. Cell wall remodeling in response to acetic acid and other weak acids is essential to protect cells from damage [28, 29]. *Atg22* deletion not only prevents cell wall composition loss, but also upregulates key genes expression of CWI pathway which may lead cell wall that is robust and compact to withstand Ac uptake. On the other hand, glucan and chitin served as an exoskeleton and a scaffold linked to the external mannoproteins which is positively related with cell resistance of adversity such as sodium houttuyfonate and caspofungin, the increase of glucan synthase likely reduces susceptibility of Ac in *S. cerevisiae* [29–34].

### Cell membrane integrity, permeability and fluidity

Cytomembrane is the permeability barrier which plays a vital role in information transfer, substance exchange and separation of cytoplasm and extracellular environment [35]. It is demonstrated that Ac enters into cytoplasm by diffusing across the cytomembrane and consequently disrupts pH homeostasis intensifying intracellular acidification which eventually induce PCD [36, 37]. Preceding researches suggest that the integrity, fluidity, and permeability of cell membrane affected by its compositions and be closely linked with the uptake as well as resistance of Ac [38–41]. We manifested that Atg22p deficiency is favorable to strengthen membrane integrity, fluidity and permeability by means of changing compositions ultimately reducing PCD induced by Ac. Phospholipids is an important structure component of membrane lipid bilayer, and make sense for membrane fluidity and integrity. Researches showed that the upregulation of phospholipids level can help *S. cerevisiae* cells to adapt Ac toxicity and decrease ROS accumulation [24, 42]. In this investigation, total content was prominently upregulated by Ac treatment which might adaptively respond to Ac stress as previously reported [42, 43]. Concretely, *atg22* deletion facilitated PG, PA accumulation while overexpression facilitated PS, PI and PE as exposed to Ac, respectively. PA is an indispensable intermediate in phospholipids metabolism and PG acts as a key fusion for two other phospholipids which may indicate that their sediment make for the decrease of membrane permeability to prevent cell from Ac diffusion [39, 42, 44, 45]. Increasing proportion of PS and PE may reduce unsaturation to impair membrane fluidity and hinder the coping with Ac exposure [40, 46]. Sterols play a crucial part in structure–function, microdomain formation, protein–lipid interactions and signal transduction [47, 48]. Sterols can increase membrane integrity by fortifying packing density and weakening acyl chain flexibility through interacting with phospholipids by which influence cell resistibility to stress [43, 49, 50]. Moreover, ergosterol is widely identified as the most important sterol associated with inhibiters resistance which can form transmembrane channel structures by interacting with some substrates and consequently boost membrane order subdued substrates permeability [51–55]. Ergosterol accumulation contributed to yeast cell survival under vanillin, ethanol, temperature, and pH stress contrasted with the disruption of ergosterol biosynthesis exacerbated the toxic effects of mono-(2-ethylhexyl)-phthalate, lovastatin, nystatin, amphotericin B, and terbinafine [38, 56–61]. Ac increased ergosterol accumulation while decreased in zymosterol which were more significant in *Atg22*Δ strain, but opposite in the overexpression strain. These data revealed that Atg22p absence promoted the transformation from zymosterol to ergosterol and amassing. Accumulation of squalene in BY4741-pESC-Atg22 strain also provided collateral evidence for *atg22* overexpression blocked ergosterol biosynthesis which possibly damages membrane permeability and exacerbates PCD induced by Ac [38, 46].

Membrane fluidity shows positive correlation with cis-unsaturated fatty acids while negative correlation with trans-unsaturated fatty acids and average length [62–64]. Data of RNA-Seq-based transcriptomics and metabolomics manifested that Ac can suppress fatty acid biosynthesis by down-regulating the expression of key genes such as *fas1, acc1 and pox1,* and strengthen fatty acid chain elongation by upregulating expression of *mct1* and *elo1* [2]. Similar results achieved in this study, demonstrating that the total content of fatty acids no matter saturated or unsaturated sharply reduced under Ac exposure. Interestingly, the deficiency of Atg22p likely promotes fatty acids synthesis which leads *Atg22*Δ strain to accumulate more fatty acids. *S. cerevisiae* increased the biosynthesis of long-chain unsaturated fatty acids and reduced the biosynthesis of short-chain fatty acids to relieve membrane disruption and integrity decrease induced by Ac [39, 40, 41]. The data indicate that *atg22* deletion may reduce the destruction of membrane structure and function as well as PCD upon harsh condition [39].

### Amino acids balance

Amino acids play essential roles in primary metabolites including protein synthesis, energy conversion, signaling pathways, therefore starvation for essential amino acids like lysine or histidine may induce severe cell stress, increased sensitivity to inhibitor and even PCD [16, 65, 66]. It is reported that Ac can repress the expression of numerous critical genes encoding protein about amino acid uptake, transportation and synthesis [5, 6, 10, 16]. Previous researches also revealed that supplementing the corresponding amino acid or upregulating expression of genes involved in sensing, signaling and uptake of amino acids increased cell survivability under Ac, for example, the presence of proline can relieve Ac toxic effect [14, 31]. To a certain degree, the maximal tolerance to Ac is dependent on a persistent and efficient capacity for acquiring available amino acids to maintain a basic physiological function in *S. cerevisiae*. Autophagy is a relatively moderate stress adaptation and self-protection tactic in extreme conditions which supplements nutrients by degrading damaged proteins as well as defective organelles in vacuoles and recycling available substances into cytoplasm to maintain cell metabolism essential for cell survival [67]. Atg22p has been proved to be a vacuolar effluxer for recycling amino acids from autophagic degradation under nitrogen starvation [15]. We found that *atg22* disruption can upregulate the expression of autophagy-related genes which may enhance autophagy. What’s more, *atg22* deletion decreased vacuole amino acid without influencing the total content. The content of cytosolic amino acid including Thr, Glu, Pru, Gly, Met, Leu, Lys, Arg all increased in *atg22*Δexcept Ala which may relieve the repression of amino acids uptake induced by Ac. Based on these results, we inferred that *atg22* deficiency may strengthen yeast cells viability under Ac pressure by promoting autophagy and amino acids fluxion from vacuole to cytoplasm which supplemented available amino acids in cytoplasm and alleviated Ac toxic effect [6]. Besides, the upregulation of *pep4*, *rgi1* and *btn2* may provide additional security for *Atg22*Δ cells [68]. To sum up, we supposed that Atg22p is the key regulator in vacuole affecting the balance of amino acids between cytosol and vacuole (Fig. 11).

### Stress sensing

The heat shock protein family has been widely reported as important regulators of apoptosis [69, 70], and allows the cells to survive under a wide variety of physiological and environmental stresses [71]. *Hsp70* displays an anti-apoptotic activity by inhibiting caspase activation and apoptosis-inducing factor (Aif1) release in mammalian cells [72, 73]. *Hsp90* played dual role of in acetic acid-induced apoptosis [74]. In addition, Fes1p promotes ubiquitin-dependent degradation of misfolded proteins interacted with *hsp70* chaperones [75]. Heat shock protein 104 (*hsp104*) is required for refolding and reactivating denatured and aggregated proteins cooperating with *hsp70/40* [76]. In *Atg22*Δ cells, *hsp104*, *hsp70* and *hsp90* chaperones are upregulated in response to Ac, which in accord with the previous study and indicate that stress-induced misfolded and damaged proteins are efficiently removed or reactivated by HSPs. This will contribute to maintaining protein homeostasis to protect cells against acetic acid attack (Fig. 13).

## Conclusions

In conclusion, we demonstrate that *atg22* deficiency protects cells from Ac attack through reducing ROS production and mitochondrial membrane potential. Overexpression of *atg22* aggravates acetic acid-induced cell death, which in turn makes yeast cells to cut down the expression of Atg22p in response to Ac. Moreover, Atg22p deficiency alters the compositions of cell wall and cytomembrane to maintain their structure and function. The *Atg22*Δ mutant accumulates high quantity of intracellular amino acids into the vacuole; a decrease of available amino acids delayed the cell death process during Ac treatment. In addition, *Atg22*Δ cells improve the transcription of genes in heat shock protein family, cell wall integrity pathway and autophagy, which protect cells against Ac stress. On the whole, this study provides new insights for the role of Atg22p in PCD induced by Ac, and presents a novel strategy to construct industrial yeast with high acid resistance for biofuel production.

## Materials and methods

### Strains and plasmids

*Saccharomyces cerevisiae* strains BY4742 (MATα *his3*Δ*1 leu2*Δ*0 lys2*Δ*0 ura3*Δ*0*) and *Atg22*Δ (BY4742 *Atg22*::KanMX4) used in this study were obtained from EUROSCARF. To construct the plasmid pESC-ATG22, the insert was amplified by PCR using genomic DNA of BY4742 as the template, ligated into the vector pESC-ura (Agilent Technologies) by homologous recombination using ClonExpress™ II One Step Cloning Kit (Vazyme Biotech, Nanjing, China). The plasmid pTEF-ATG22-EGFP was constructed from pTEF2 by ClonExpress MultiS One Step Cloning Kit (Vazyme Biotech, Nanjing, China), with genomic DNA of BY4742 and the plasmid p416ADH-PEP4-GFP (a gift from Dr. Vítor Costa) as the templates. The wild-type BY4742 was transformed with the empty vector (pESC-ura) and pESC-ATG22 for comparison of *atg22* overexpression in yeast. And the wild-type strain was transformed with pTEF-ATG22-EGFP for Atg22p tagging, and the empty vector pTEF2 served as control. All strains were transformed by the lithium acetate method, and validated by PCR analysis and gene sequencing. The primers and restriction enzymes for the recombinant plasmids are listed in Additional file 1: Table S5.

### Yeast growth and acetic acid treatment

All yeast strains were grown in synthetic complete medium [SC; 2% (w/v) d-glucose, 0.67% (w/v) yeast nitrogen base without amino acids, 0.008% (w/v) histidine, 0.02% (w/v) leucine, 0.003% (w/v) lysine and 0.032% (w/v) uracil] to exponential phase in an orbital shaker, at 28 °C and 180 rpm. The strains carrying plasmids were grown in the same medium, but without uracil. For yeast cells harboring pESC-ura or pESC-ATG22, 2% (w/v) galactose was used for the induction of target protein expression instead of 2% (w/v) d-glucose. For all experiments treated with acetic acid, yeast strains were grown under the above conditions until exponential phase, collected and resuspended with a final cell concentration of 1 × 10^7^ cells/ml in fresh SC or SG broths at pH 3.0 (set with HCl) containing 150 mM acetic acid, and incubated at 28 °C in an orbital shaker at 160 rpm, with a ratio of flask volume/medium of 5:1. The control groups were treated without Ac under same culture conditions. Growth curves of BY4742 and *Atg22*Δ were calculated by measuring OD_600_.

### Cell viability assay

Both WT and mutant cells in exponential phase were treated with different concentrations of acetic acid (50, 100 and 150 mM) for 2 h in SC media (pH 3.0). Then, cell numbers were calculated, and 400 cells in each independent experiment were spread on YPD [1% (w/v) yeast extract, 2% (w/v) peptone, 2% (w/v) glucose and 2% (w/v) agar] agar plates. Cell viability was measured by counting colony-forming units (CFU) after 3 days of incubation at 30 °C.

### Annexin V/PI costaining and DHE staining

Phosphatidylserine externalization and loss of plasma membrane integrity were detected by flow cytometry (FC500MCL, Beckman Coulter, Brea, USA), using the Annexin V/PI apoptosis kit (Lianke, Hangzhou, China). Before that, cells were harvested and washed in sorbitol buffer (1.2 M sorbitol, 0.5 mM MgCl_2_, 35 mM K_2_HPO_4_, pH 6.8), digested with 20 U/ml lyticase (Sigma-Aldrich) at 30 °C for 50 min. Subsequently, cells were washed and resuspended in 500 μl of binding buffer (1.2 M sorbitol, 10 mM HEPES/NaOH, 140 mM NaCl, 2.5 mM CaCl_2_, pH 7.4), then incubated with Annexin V and PI for 10 min at room temperature in the dark.

To quantify intracellular ROS, cells were firstly harvested and resuspended in 500 μl PBS and stained with 5 μg/ml dihydroethidium (DHE) (Cayman Chemical, Ann Arbor, USA) for 30 min in the dark at room temperature [77]. Cells with red fluorescence [FL-3 channel (488/620 nm)] were detected using the Cytomics FC500MCL flow cytometer (Beckman Coulter).

### Atg22p tagging and PI staining

For Atg22p labeling, cells carrying the plasmid pTEF-ATG22-EGFP were observed using a Zeiss LSM 780 confocal microscope (Carl Zeiss MicroImaging, Göttingen, Germany). The strains were grown and treated with or without acetic acid (150 mM, pH 3.0) for different times as described in the above conditions, then immobilized in the slides prior to confocal microscopy. Propidium iodide (PI) (Lianke, Hangzhou, China) staining was used to analyze the relevance between Atg22p expression and acetic acid-induced cell death. In addition, the positive cells stained by PI and cells with GFP fluorescence were quantified by flow cytometry.

### Morphology observation

Scanning electron microscopy (SEM) and transmission electron microscopy (TEM) were used to observe the surface and interior structure, respectively, as previous studies noted. In short, after double fixation, dehydration SEM samples were coated with gold–palladium in Hitachi Model E-1010 ion sputter for 4–5 min and observed in Hitachi Model SU-8010 SEM. After pretreatment the steps consisted of double fixation, dehydration, infiltration, Spurr resin embedding and ultrathin section. Sections of TEM specimen were stained and observed in Hitachi Model H-7650 TEM.

### Extraction and measurement of cell wall polysaccharides by high-performance liquid chromatography (HPLC)

All experimental strains were treated with different concentrations of acetic acid (0 and 150 mM) for 2 h after reaching exponential phase in SC media (pH 3.0), harvested and washed thrice with distilled water by resuspension and centrifugation at 12,000×*g* for 5 min at 4 °C. Cells were collected and broken by ultrasonic cell disruptor completely. After twice centrifugation and resuspension, the underlayer deposition containing cell wall was gathered and freeze-dried. About 20 mg specimen was treated with 150 μl 72% (W/V) concentrated sulfuric acid overnight and boiled 4 h. Saturated Ba(OH)_2_ added to terminated reaction until reaching neutral pH. Supernatant was injected into HPLC (Waters E2695, USA) for detection after centrifugation at 12,000×*g* for 5 min at 4 °C and filtration of 0.22 μ membrane as previously described.

### Measurement of fluorescence anisotropy

1,6-Diphenyl-1,3,5-hexatriene (TMA-DPH) was used as probe to detect the membrane fluidity of *Saccharomyces* *cerevisiae* cells in logarithmic phase as previous study described. In brief, all strains were treated with acetic acid (150 mM, pH 3.0) or equal volume PBS for 120 min after reaching exponential phase cultured in SC media (pH 3.0) and reacted with 2 μl probe (1 mM) 10 min in dark situation. A spectrofluorimeter (Photon Technology International, Princeton, NJ, USA) with excitation at 360 nm and emission at 450 nm was applied to measure the steady-state fluorescence anisotropy. The fluorescence anisotropy value (*r*) was calculated using the following formula:

$$r = \, \left( {I_{\text{VV}} - GI_{\text{VH}} } \right)/\left( {I_{\text{VV}} + GI_{\text{VH}} } \right),\;\;\;G = I_{\text{HV}} /I_{\text{HH}} ,$$

where *I* represents the correlation factor for instrument polarization and fluorescence intensity; VV indicates the measurement of both polarizers positioned vertically, the opposite of HH; VH indicates the measurement of excitation in vertical emission polarizers horizontal positions which is opposite to HV. Triplicate treatment implemented during all tests and negative correlation showed between *r* and membrane fluidity.

### Extraction and measurement of membrane fatty acid by gas chromatography (GC)

Cells were freeze-dried after cultivation, treatment, and collection implemented as above. About 50 mg samples were used for extraction and methyl esterification of total lipids before gas chromatography detection was carried out. By passage through a polyethylene glycol capillary column, components were separated and calculated as previously study described.

### Membrane sterol extraction and measurement by GC–mass spectrometry (GC–MS)

The cultivation, treatment, collection and lyophilization of *Saccharomyces* *cerevisiae* cells were implemented as above. After 40 μl cholesterol (0.5 mg/ml) was added into 50 mg freeze-dried samples as an internal standard sterol, all pretreatment processes and detection methods of gas chromatograph–mass spectrometer were operated as previous described.

### Membrane phospholipid extraction and measurement by LC–mass spectrometry (LC–MS)

Freeze-dried samples were obtained as described above, 55 mg dried specimen was weighed and used for phospholipid extraction. After six steps contained chloroform–methanol (2:1, vol/vol) treatment, vortex, ultrasound, organic phase collection, and dried under a nitrogen stream. Then, phospholipid extractive dissolved in chloroform–methanol (1:1, vol/vol) and analyzed by electrospray ionization mass spectrometry (UPLC-Xevo TQ MS, USA) as previous described.

### Measurement of intracellular amino acids

Before amino acids determination, 100 mg fresh yeast cells were collected and washed by centrifugation after treatment with 150 mM acetic acid (pH 3.0) for 120 min, and frozen in liquid nitrogen. The equivalent cells of BY4742 and *Atg22*Δ with acetic acid treatment were used for extraction of vacuolar and total amino acid pools by the Cu^2+^ method as given in references [18, 78, 79]. In brief, the collected cells were washed twice with distilled water and incubated in Buffer A (2.5 mM potassium phosphate buffer, pH 6.0, 0.6 M sorbitol, 10 mM glucose, and 0.2 mM CuC1_2_) at 30 °C for 10 min. Then cell suspensions were filtered with 0.45-μm membrane filters (Millipore) and washed 5 times with Buffer A without 0.2 mM CuCl_2_. The retained cells were resuspended in distilled water and boiled for 15 min. The supernatant was subsequently collected as the vacuolar extract after centrifugation at 25,000×*g* for 3 min. For total intracellular extracts, yeast cells were washed twice with Buffer A without 0.2 mM CuCl_2_, suspended in distilled water followed with same procedures as above. These extracts were analyzed using a Hitachi L-8900 amino acid analyzer (Hitachi, Tokyo, Japan).

### RNA isolation and RT-qPCR

Total RNA was extracted from yeast cells using the E.Z.N.A.^®^ Yeast RNA Kit (OMEGA Bio-tek, Norcross, USA), following the manufacturer’s protocols. The genomic DNA contamination was eliminated and total RNA was reverse-transcribed to cDNA using the HiScript^®^ Q RT SuperMix for qPCR (+gDNA wiper) (Vazyme Biotech, Nanjing, China). For quantitative real-time (qPCR) analysis, 30 ng of cDNA was analyzed with ChamQ SYBR qPCR Master Mix (Vazyme Biotech, Nanjing, China) according to the manufacturer’s instructions, using CFX Touch™ Real-Time PCR Detection System (Bio-Rad, Hercules, USA). Relative quantification was performed by the 2^−ΔΔCt^ method. ACT1 gene was used to quantify the mRNA levels of all genes. All primers for qPCR were designed using Primer Premier 6.0 software (PREMIER Biosoft International), and the sequences were included in Additional file 1: Table S6. Gene-specific amplification was verified by melting curve analysis and agarose gel electrophoresis.

## Supplementary information

**Additional file 1.** Additional figure and tables.

## Data Availability

The data sets analyzed during the current study are available from the corresponding author on reasonable request.

## Abbreviations

PCD: programmed cell death
Ac: acetic acid
HMF: hydroxymethyl furfural
ATP: adenosine triphosphate
RT-qPCR: real-time quantitative polymerase chain reaction
CFU: colony-forming units
WT: wild-type
OD 600: optical density at 600 nm
SC media: 2% (w/v) d-glucose, 0.67% (w/v) yeast nitrogen base without amino acids, 0.008% (w/v) histidine, 0.02% (w/v) leucine, 0.003% (w/v) lysine and 0.032% (w/v) uracil
SG media: 2% (w/v) galactose, 0.67% (w/v) yeast nitrogen base without amino acids, 0.008% (w/v) histidine, 0.02% (w/v) leucine, 0.003% (w/v) lysine and 0.032% (w/v) uracil
LB medium: 0.5% yeast extract, 1% tryptone and 1% NaCl
YPD medium: 1% yeast extract, 2% peptone and 2% glucose
PCR: polymerase chain reaction
DCW: dry cell weight
PI: propidium iodide
ROS: reactive oxygen species
DHE: dihydroethidium
EGFP: enhanced green fluorescent protein
MFI: mean fluorescence intensity
SEM: scanning electron microscopy
TEM: transmission electron microscopy
CWI pathway: cell wall integrity pathway
PG: phosphatidyl glycerol
PA: phosphatidic acid
PS: phosphatidylserine
PI: phosphatidylinositol
PE: phosphatidylethanolamine
PC: phosphocholine
MEHP: mono-(2-ethylhexyl)-phthalate
HPLC: high-performance liquid chromatography
TMA-DPH: 1,6-diphenyl-1,3,5-hexatriene
GC: gas chromatography
GC–MS: GC–mass spectrometry
LC–MS: LC–mass spectrometry
