# Supplementary material for: Deletion of Atg22 gene contributes to reduce programmed cell death induced by acetic acid stress in Saccharomyces cerevisiae
Source: Biotechnol Biofuels. 2019 Dec 27;12:298. doi: 10.1186/s13068-019-1638-x (PMC6933646; doi:10.1186/s13068-019-1638-x)
Supplement: Supplementary file 1 — Additional file 1. Additional figure and tables. [file 13068_2019_1638_MOESM1_ESM.docx]

**Additional file 1**

Figure S1. Plasmid profile of pESC-ATG22 and pTEF-ATG22-EGFP

**Table S1.** Fatty acids content in cell membrane.

|  | Content (μg/g) | | | | | |  |
| --- | --- | --- | --- | --- | --- | --- | --- |
|  | Control | | | 150 mM Ac | | | |
| Fatty acid name | BY4742-  pESC-ura | Atg22Δ-  pESC-ura | BY4742-  pESC-Atg22 | BY4742-  pESC-ura | Atg22Δ-  pESC-ura | BY4742-  pESC-Atg22 | |
| Myristic acid (C14:0) | 0.68±0.04 | 2.34±0.98 | 1.4±0.07 | 0.52±0.18 | 1.19±0.003 | 1.01±0.26 | |
| Palmitic acid (C16:0) | 22.16±0.55 | 29.86±3.54 | 18.68±0.006 | 18.0216±0.06^b^ | 23.66±1.76^a^ | 24.58±2.58^a^ | |
| Stearic acid (C18:0) | 5.66±0.02 | 4.31±0.68 | 5.25±0.007 | 4.74±0.063 ^b^ | 7.28±0.16 ^a^ | 6.83±0.78 | |
| Lignoceric acid (C24:0) | 2.4±0.17 | 2.41±0.97 | 1.79±0.69 | 4.13±0.97 | 21.27±7.66 | 14.05±2.56 ^a^ | |
| Palmitoleic acid (C16:1) | 65.51±4.7 | 77.78±2.15 | 42.85±0.97 | 36.85±0.54 ^b^ | 56.06±2.37 ^b^ | 53.86±6.91 | |
| Elaidic acid (C18:1) | ND | ND | 26.41±0.57 | 19.13±2.37 ^b^ | 35.79±2.42 ^b^ | 26.36±2.95 | |
| Oleic acid (C18:1) | 40.4±13.18 | 38.88±12.36 | 1.6±0.15 | 1.51±0.2492 ^a^ | 1.83±0.5 ^a^ | 2.05±0.18 | |
| Linolelaidic acid (C18:2) | 2.18±0.05 | 1.96±0.15 | 1.6±1.08 | ND^c^ | ND^b^ | ND | |
| Caprylic acid (C8:0) | 0.68±0.12 | 1.38±0.01 | 1.72±0.22 | 1.3±0.53 | 1.85±0.52 | 1.55±0.1 | |
| Capric acid (C10:0) | 1.69±1.98 | 1.59±0.22 | 1.61±0.26 | 1.52±0.74 | 2.32±0.09^a^ | 1.23±0.12 | |
| Undecanoic acid (C11:0) | 0.28±0.1 | 0.23±0.08 | 0.25±0.03 | ND | 0.28±0.08 | 0.22±0 | |
| Lauric acid (C12:0) | 0.8±0.13 | 3.05±2.15 | 1.38±0.03 | 0.5±0.16 | 1.63±0.1 | 1.17±0.31 | |
| Myristoleic acid (C14:1) | 0.19±0.01 | 0.59±0.11 | 0.27±0.09 | ND | 0.36±0.04 | 0.34±0.07 | |
| Pentadecanoic acid (C15:0) | 0.63±0.11 | 0.84±0.41 | 0.3±0.1 | 0.34±0.03 | 0.24±0.04 | 0.37±0.04 | |
| cis-10-Pentadecenoic acid (C15:1) | 0.19±0.03 | 0.28±0.14 | ND | ND | 0.29±0.01 | 0.26±0.03 | |
| Heptadecanoic acid (C17:0) | 0.36±0.05 | ND | 0.22±0.01 | 0.22±0.01^a^ | ND | 0.29±0.1 | |
| cis-10-Heptadecenoic acid (C17:1) | 0.34±0.02 | ND | 0.19±0.07 | 0.59±0.52 | 0.49±0.18 | 0.45±0.13 | |
| Linoleic acid (C18:2) | 0.3±0.21 | 0.13±0.01 | 0.24±0.04 | ND | 0.15±0.03 | 0.46±0.47 | |
| cis-4,7,10,13,16,19-Docosahexaenoic  acid (C22:6) | 1.1±0.05 | 0.78±0.02 | 1.07±0.23 | 0.22±0.31 | 0.81±0.21 | 0.95±0.01 | |

The data represent three independent experiments and ND means no detected in this sample. In addition, a, b, c represents ***p***≤0.05，***p***≤0.01，***p***≤ 0.001 respectively determined by the t test.

**Table S2.** Sterols content in cell membrane.

|  | Content (μg/g) | | | | | | |
| --- | --- | --- | --- | --- | --- | --- | --- |
|  | Control | | | 150 mM Ac | | | |
| Sterol name | BY4742-  pESC-ura | Atg22Δ-  pESC-ura | BY4742-  pESC-Atg22 | BY4742-  pESC-ura | Atg22Δ-  pESC-ura | BY4742-  pESC-Atg22 |  |
| Squalene | 70.09±0.31 | 76.86±0.25 | 72.43±1.83 | 75.36±0.66 ^c^ | 67.53±1.89 ^c^ | 80.34±0.8 ^b^ |  |
| lanosterol | 89.03±0.52 | 92.14±1.3 | 80.87±4.4 | 90.66±0.35 ^b^ | 95.63±1.81 ^a^ | 88.42±0.69^a^ |  |
| 4,4-Dimethylzymosterol | 146.63±3.58 | 154.08±1.4 | 138.3±9.43 | 156.63±2.63 ^b^ | 153.87±1.34 | 166.78±1.73 ^b^ |  |
| Zymosterol | 134.85±1.03 | 137.58±5.92 | 133.27±1.51 | 129.73±0.73 ^b^ | 126.92±2.05 ^a^ | 142±0.26 ^c^ |  |
| Fecosterol | 48.07±2.36 | 48.87±1.01 | 45.76±1.01 | 48.38±0.96 | 50.87±1.34 | 53.86±1.42 ^b^ |  |
| Ergosterol | 601.92±2.36 | 592.98±16.92 | 604.53±9.2 | 615.54±7.7 ^a^ | 636.2±10.42 ^a^ | 597.68±8.01 |  |

The data represent three independent experiments, and ND means no detected in this sample. In addition, a, b, c represents ***p***≤0.05, ***p***≤0.01, ***p***≤ 0.001 respectively determined by the t test.

**Table S3.** Phospholipids content in cell membrane.

|  | Content (μg/g) | | | | | | |
| --- | --- | --- | --- | --- | --- | --- | --- |
|  | Control | | | 150 mM Ac | | | |
| Phospholipids name | BY4742-  pESC-ura | Atg22Δ-  pESC-ura | BY4742-  pESC-Atg22 | BY4742-  pESC-ura | | Atg22Δ-  pESC-ura | BY4742-  pESC-Atg22 |
| PA | 2.99±0.26 | 13.18±0.24 | 2.733±0.09 | 7.88±0.37^c^ | 9.09±0.14 ^c^ | | 3.34±0.05 ^c^ |
| PG | 8.13±0.13 | 3.98±0.12 | 9.29±0.21 | 7.08±0.08 ^c^ | 16.6±0.9 ^c^ | | 15.2±0.27 ^c^ |
| PC | 2984.77±14.31 | 2704.62±33.83 | 2802.24±85.34 | 3156.5±49.84 ^c^ | 2905.4±33.69 ^c^ | | 3111±101.69 ^c^ |
| PE | 694.13±7.41 | 707.09±3.96 | 636.22±7.99 | 1911.24±10.79 ^c^ | 2024.53±17.93 ^c^ | | 2251.76±45.35 ^c^ |
| PI | 142.56±2.83 | 123.8±0.83 | 120.04±0.88 | 152.29±1.74 ^b^ | 144.58±0.57 ^c^ | | 586.31±11.04 ^c^ |
| PS | 38.26±0.78 | 46.81±0.35 | 42.37±0.66 | 40.82±0.19 ^b^ | 44.7±0.28 ^c^ | | 47.14±0.26 ^c^ |

The data represent three independent experiments and ND means no detected in this sample. In addition, a, b, c represents ***p***≤0.05, ***p***≤0.01, ***p***≤ 0.001 respectively determined by the t test

**Table S4.** Functional categories and comparative expression fold changes of key genes in *S. cerevisiae* BY4742 and *Atg22*Δ strains after treatment with 150 mM Ac for 120 min.

| Gene and category | Fold change | *P* value | Function description |
| --- | --- | --- | --- |
| **Heat shock protein family** | | | |
| *ssa3** | 3.245±0.085 | 0.000007 | Hsp70 chaperone |
| *fes1** | 2.874±0.113 | 0.000009 | Hsp70 (Ssa1p) nucleotide exchange factor |
| *hsp30** | 2.332±0.146 | 0.000105 | Heat shock protein 30 |
| *ssa4** | 2.037±0.088 | 0.000035 | Hsp70 chaperone |
| *hsp82** | 1.983±0.089 | 0.000083 | Hsp90 chaperone |
| *hsp104** | 1.876±0.099 | 0.000267 | Heat shock protein 104 |
| *cpr6** | 1.619±0.083 | 0.000341 | Hsp90 cochaperone |
| *hsc82** | 1.543±0.064 | 0.000234 | Hsp90 chaperone |
| *sti1** | 1.502±0.148 | 0.004655 | Hsp70/90 cochaperone |
| **Cell wall integrity pathway** | | | |
| *slt2** | 2.190±0.235 | 0.001113 | Serine/threonine MAP kinase |
| *gsc2** | 2.168±0.073 | 0.000013 | Catalytic subunit of 1,3-beta-glucan synthase |
| *pkc1** | 2.068±0.105 | 0.000690 | Protein serine/threonine kinase |
| *tip1** | 1.626±0.057 | 0.000102 | Major cell wall mannoprotein with possible lipase activity |
| *rlm1** | 1.532±0.113 | 0.006070 | MADS-box transcription factor |
| *rho1* | 1.351±0.069 | 0.042904 | GTP-binding protein of the rho subfamily of Ras-like proteins |
| *ptp2* | 1.347±0.066 | 0.001224 | Nuclear phosphotyrosine-specific phosphatase |
| *mkk2* | 1.324±0.064 | 0.013174 | MAPKK involved in the protein kinase C signaling pathway |
| *zeo1* | 1.152±0.040 | 0.002794 | Peripheral membrane protein of the plasma membrane |
| *bck1* | 1.146±0.096 | 0.228685 | MAPKKK acting in the protein kinase C signaling pathway |
| **Autophagy** | | | |
| *atg8** | 2.442±0.126 | 0.000061 | Component of autophagosomes and Cvt vesicles |
| *atg12** | 2.095±0.124 | 0.000206 | Ubiquitin-like modifier involved in autophagy and the Cvt pathway |
| *atg2** | 2.039±0.306 | 0.004699 | Peripheral membrane protein required for autophagic vesicle formation |
| *atg11* | 1.489±0.083 | 0.004972 | Involved in cytoplasm to vacuole transport (Cvt), pexophagy, mitophagy and nucleophagy |
| *atg1* | 1.466±0.085 | 0.000984 | Protein serine/threonine kinase |
| *vps30* | 1.455±0.071 | 0.000498 | Subunit of phosphatidylinositol (PtdIns) 3-kinase complexes I and II |
| *atg7* | 1.328±0.228 | 0.070621 | Autophagy-related protein and dual specificity member of the E1 family |
| *atg32* | 1.281±0.011 | 0.000185 | Mitochondrial outer membrane protein required to initiate mitophagy |
| *atg18* | 0.936±0.069 | 0.329938 | Phosphoinositide binding protein required for vesicle formation in autophagy and the Cvt pathway |
| *atg13* | 0.912±0.046 | 0.250548 | Regulatory subunit of the Atg1p signaling complex |
| *atg16* | 0.832±0.022 | 0.030293 | Conserved protein involved in autophagy |
| *atg22* | - | - | Vacuolar integral membrane protein required for efflux of amino acids |
| **Histone acetylation and deacetylation** | | | |
| *epl1* | 1.214±0.030 | 0.001573 | Subunit of the histone acetyltransferase complex (NuA4) |
| *set2* | 1.177±0.011 | 0.000036 | Histone methyltransferase with a role in histone deacetylation |
| *esa1* | 1.170±0.066 | 0.013079 | Catalytic subunit of NuA4 |
| *hos1* | 1.170±0.070 | 0.103657 | Class I histone deacetylase (HDAC) family member |
| *hst3* | 1.109±0.099 | 0.328372 | Member of the Sir2 family of NAD(+)-dependent protein deacetylases |
| *ada2* | 1.051±0.076 | 0.421319 | Component of the ADA and SAGA transcriptional adaptor/HAT (histone acetyltransferase) complexes |
| *sgf29* | 1.029±0.076 | 0.699799 | Component of the HAT/Core module of the SAGA, SLIK, and ADA complexes |
| *ahc2* | 1.016±0.060 | 0.760529 | Component of the ADA histone acetyltransferase complex |
| *hpa2* | 0.998±0.039 | 0.924951 | Tetrameric histone acetyltransferase |
| *yaf9* | 0.844±0.052 | 0.049649 | Subunit of NuA4 histone H4 acetyltransferase and SWR1 complexes |
| *acc1* | 0.800±0.031 | 0.001706 | Acetyl-CoA carboxylase |
| **Vacuolar degradation** | | | |
| *pep4** | 1.528±0.035 | 0.000025 | Vacuolar proteinase A |
| *rny1* | 1.308±0.030 | 0.000154 | Vacuolar RNase of the T(2) family |
| *prb1* | 0.966±0.168 | 0.728684 | Vacuolar proteinase B |
| **Other genes** | | | |
| *rgi1** | 2.041±0.056 | 0.000005 | Uncharacterized protein induced by respiratory conditions |
| *btn2** | 1.577±0.073 | 0.000473 | v-SNARE binding protein |
| *rad53* | 1.157±0.029 | 0.010629 | DNA damage response protein kinase |
| *rsp5* | 1.024±0.029 | 0.560384 | E3 ubiquitin ligase of NEDD4 family |
| *tor2* | 0.885±0.077 | 0.144288 | PIK-related protein kinase and rapamycin target |
| *vma1* | 0.833±0.125 | 0.110168 | Subunit A of the V1 peripheral membrane domain of V-ATPase |

Data represent means±SD for the relative gene expression (150 mM acetic acid-treated *Atg22*Δ log-phase cells/BY4742 log-phase cells normalized to *Act1* expression).

* Genes showing significantly enriched transcription abundance in *atg22*Δ, with the expression fold changes greater than 1.5-fold (n=3; *P*< 0.01) compared with the wild type control.

**Table S5**. Primers and restriction enzymes for the recombinant plasmids used in this study.

| Plasmid | Primer | Restriction enzyme |
| --- | --- | --- |
| pESC-Atg22 | AACCCTCACTAAAGGGCGGCCGCATGAGCTATGGAACTATAAATGA(forward)  CCTTGTAATCCATCGATACTAGTGAATCCAACCTTCTTTCACTTT (reverse) | *Not* I  *Spe* I |
| pTEF-Atg22-EGFP | CCCCATCGATCACCCACTAGTATGAGCTATGGAACTATAAATGA(forward)  TGAAGAACCACTACCGGATCCATCCAACCTTCTTTCACTTTC (reverse)  GGTAGTGGTTCTTCAGCTAGCATGGTGAGCAAGGGCG (forward)  TTTAAAGCTTGGGGGGAATTCTTACTTGTACAGCTCGTCCAT (reverse) | *Spe* I  *BamH* I  *Nhe* I  *EcoR* I |

**Table S6.** Primers used in qPCR analysis

| Gene | Forward primer (5' → 3') | Reverse primer (5' → 3') | Length (bp) |
| --- | --- | --- | --- |
| *act1* | TCCCAGGTATTGCCGAAAGAATGC | GCCAAGATAGAACCACCAATCCAGA | 124 |
| *esa1* | TGGTCGGACACTCTCATAACGCTAT | GCTGACCCTTGTAATATCGCAGGAT | 145 |
| *btn2* | TTCCGAAGGTGGCATCAACGAAC | CGCTTTCTCCGCTTCTTCCTCCT | 115 |
| *hsp30* | ATGCTACGGACGATGTGGAAGATG | GCTTGCTCTGCTTCAGGTTCGG | 115 |
| *rgi1* | CCCACCATTTGTGCTACACGAGTC | TCAGGTTTCCTAACGGCTTGCTTAA | 150 |
| *zeo1* | GCTACTCCAGAAGCTGAACAGGTGA | TTGGTTTCCTTCTTCTCACTGACGG | 143 |
| *epl1* | GCATCCTCTTCTTCAACATCACAGC | AGAGCATCCACGTCTTCCAATACAA | 101 |
| *vma1* | GCTACCTACCAGACTTACGCTCCAA | TGTCAGACAATCCATCACCAATCCA | 148 |
| *gsc2* | TTGCTGACCCGTGAGTTCAAGAATG | CCTTGTCGGTTGAGTCCATGCCATA | 105 |
| *ptp2* | TCCGCATAATGGTGACCTTACTTCC | TCTACGCCGCATGAATCTAACCAAT | 102 |
| *rad53* | GTTGCTGCTCATGGTGCGGTTG | GGGCTTTAGGTCACGATGGCTGAT | 114 |
| *atg8* | AAGGCGGAGTCGGAGAGGATTG | CCTACGGTAAGGTCAGCAGGAACT | 131 |
| *atg11* | GGGAAAGAATGGTTTGTAGGCAGAA | CCACAACCGCAGTGACCTGAA | 121 |
| *atg32* | CACGATTCCGCAACATTCCC | TTGCCCAGGCTGGCAGATC | 139 |
| *rsp5* | CCTGGGATGACCCAAGACT | CGGGCTGGGACCTGAAAT | 99 |
| *acc1* | TTCCACCTCAGTTAATTCGCCGTTT | TTATTCCGCCAAGCATCGTCATCC | 123 |
| *tip1* | GGATGGAGCGGCAGAGGATGTA | CTGCTGCTCTTGCCTCCGTTTC | 116 |
| *fes1* | CTCGTCAGACAAGCACTCAATCGTA | TGAGAGCCATTGCCTTATTGACCG | 119 |
| *hos1* | TTCCCTACGCCAGAAAGGATGATC | GCCAATTCACTCAACTCACTCCATT | 148 |
| *hst3* | GCGTAACCAAGCAGTTGAGCAGAT | GTAGAGCCCATCAGAAGAGCGAAAG | 107 |
| *set2* | CGCCGCCTGATGACTTAACCAA | TGCTGATGATAATGCCAACGCCTT | 119 |
| *ada2* | TACAGGAAAGCATCGCCCTTACCA | GCCAATTACCGAGCCCTAAAGTCTG | 137 |
| *ahc2* | AACAACGAAGAACACAGCTAGAGAC | TCCGCCAATACATCAACAAATTCCT | 127 |
| *hpa2* | GCGGGCGGCAAACTAATCCA | CCGACCTTGACGTATAGCAGCT | 119 |
| *sgf29* | GACGGCGAATGGATACAGTGTGAA | TGGCGGGATCAACAGTAGTTCTTTC | 144 |
| *yaf9* | TGCTCCTGCCGAACATACTCATT | ATGGATCTCACAGGGTTTGGGTAG | 129 |
| *atg22* | TTTCGTTGGGATTTGGTGGTTTGTG | CTGCGGGCGGATGCTAATGTAG | 108 |
| *pep4* | GCCATACGATTACACGCTTGAAGTT | CGCAAGAAGGCATCACCAACGA | 111 |
| *atg1* | GACGCTTGTCTATATCCTCGCTGAA | TGATTTGTTGCTGTTGCTGCTGTTG | 114 |
| *atg2* | GAAGGGCATTTGGGAAGGAGTCTGA | GTGTTCTCCAATATCGCCTGTGTCC | 122 |
| *atg7* | AACGAGGAGGCTCAGCATAAGGAT | TCAATAACGACGGAAGCCATCTGC | 115 |
| *atg12* | CCACAAATAATGGAACGGCAATGGA | CAGAAATGTCACTCGCCAAACCAAG | 135 |
| *atg13* | CCAGAATCAGCCAGAACAAGAAGGA | GGTGTTGTCTCTGTTGCTGTTGCT | 125 |
| *atg16* | ACGGAAAGGTTGAATGACGAATTGA | CGCTGTTCATGGCTTCTGTCTCTT | 148 |
| *atg18* | GGTGGCAACACAGAGACCAGTTTCA | GCTGTTACCGCCGTTACTGCTTGT | 135 |
| *hsc82* | AAGAATACGAACCATTGACCAAGGC | CAGACCAGCCGAATTGACCAGTT | 126 |
| *hsp82* | TCTATCTGCTGGTGCCGATGTATCC | TGTATTGTTCGTCGTCGTTGCTCTT | 119 |
| *hsp104* | CCGTCGCAAGAGATTCTAAGCCAGA | GTGGTGGAGTCGGCATCTTCATCT | 112 |
| *cpr6* | ACACCTCATTTGGACGGGAAGCA | GGTAACACGCCACAGTCATCAATCT | 146 |
| *sti1* | AGGAATCCGAGCCAATGGAAGTTGA | GCAGTTCCCACGCCTTGTTGTAGT | 138 |
| *ssa3* | GGCTACCGCAGGAGACACTCATT | GCCGCAGTTCTCAATCTTCTTAACG | 141 |
| *ssa4* | ACAAGAAATCGCAGAAGGAGCACAA | TCACCAGCAGTAGCCTTAACCTCAA | 118 |
| *rny1* | AGCAAGATACAGAGGAGTCGTTCGT | TCGTAATTCGCTGGTGTACCTTGAC | 114 |
| *vps30* | CGGTGATTGGTTGATTCTGCCTGTC | GCTATCGTAGAAAGCTGCCGAGTGA | 144 |
| *prb1* | CATCTCTGCTGCTTTGGTCATCCC | TGGCTCCTCATCATCGTCCTTAGTG | 127 |
| *rlm1* | AACTCCGCAACAGCAACAGCAG | TCGTGGAAGGAGAAGATGAACTGGA | 101 |
| *slt2* | TGTGAGACAACCGCTATTAGAAGAG | GCGTTGCCATTATCCACATCTGAA | 117 |
| *mkk2* | CGTAGACCAGTTAGAGGAAGAAGCA | AATCGGCACTTGGCTACAGAACC | 111 |
| *bck1* | CATCTTCATCACCACCGCCAATACC | GCACTGGAACTGTCTTGTCTGGATT | 150 |
| *pkc1* | GCGTGAAACCACCCTACATCCC | CTCTTCTTGCTGGCTCGTTGTCA | 131 |
| *rho1* | CCGTTACATCACAGGAGGGACAATC | GCCTCAAACACTTCTCTGACACCAT | 106 |
| *tor2* | GCTGGCTGCTTCCTCTGGTTATGT | ACGAACAGTTCCACGCCTGATATGA | 121 |
